# A systematic review of passive data for remote monitoring in psychosis and schizophrenia

**DOI:** 10.1038/s41746-025-01451-2

**Published:** 2025-01-27

**Authors:** Siân Bladon, Emily Eisner, Sandra Bucci, Anuoluwapo Oluwatayo, Glen P. Martin, Matthew Sperrin, John Ainsworth, Sophie Faulkner

**Affiliations:** 1 Centre for Health Informatics, Division of Informatics, Imaging and Data Science, School of Health Sciences, Faculty of Biology, Medicine and Health, The University of Manchester, Manchester, M13 9PL UK; 2 Division of Psychology and Mental Health, School of Health Sciences, Faculty of Biology, Medicine and Health, Manchester Academic Health Science Centre, The University of Manchester, Manchester, M13 9PL UK; 3 Greater Manchester Mental Health NHS Foundation Trust, Manchester, UK; 4 NIHR Manchester Biomedical Research Centre, Manchester University Hospitals NHS Foundation Trust, Manchester Academic Health Science Centre, Manchester, UK

**Keywords:** Translational research, Health care

## Abstract

There is increasing use of digital tools to monitor people with psychosis and schizophrenia remotely, but using this type of data is challenging. This systematic review aimed to summarise how studies processed and analysed data collected through digital devices. In total, 203 articles collecting passive data through smartphones or wearable devices, from participants with psychosis or schizophrenia were included in the review. Accelerometers were the most common device (*n* = 115 studies), followed by smartphones (*n* = 46). The most commonly derived features were sleep duration (*n* = 50) and time spent sedentary (*n* = 41). Thirty studies assessed data quality and another 69 applied data quantity thresholds. Mixed effects models were used in 21 studies and time-series and machine-learning methods were used in 18 studies. Reporting of methods to process and analyse data was inconsistent, highlighting a need to improve the standardisation of methods and reporting in this area of research.

## Introduction

Psychosis is a collection of experiences that involve an individual losing some contact with reality. It can involve seeing or hearing things others cannot see or hear (hallucinations), believing things that are not shared by others (delusions), and/or confused thinking and speech^1^. Schizophrenia is considered a severe mental illness and is a diagnostic label given to an individual when symptoms of psychosis occur over a certain period of time, at a certain frequency, and cause significant impairment. The experience of psychosis can be costly to health services and have long-lasting effects on individuals^2–5^. Relapse of psychosis is common^6^. Although there is no universally accepted definition of relapse, it typically refers to the return or exacerbation of psychotic symptoms following a period of improvement or stability that results in a significant change in clinical management^7^.

Monitoring people with psychosis and schizophrenia and identifying relapse in a timely manner is challenging as contact with mental health services can be infrequent, and retrospective recall of thoughts, feelings and symptoms can lack specificity and accuracy^8^. With advancements in digital technologies, there is increasing focus on using sensors in digital tools such as mobile phones and wearable devices to support real-time passive monitoring of people with psychosis and schizophrenia. Digital technology encompasses a wide range of devices and applications that process, transmit, and store data. Examples include smartphones, computers, and smartwatches. For many years, research-grade devices, such as accelerometers and actigraphs (which measure movement), have been used in studies to collect physiological and behavioural data from people with psychosis remotely^9^. More recently, there has been a shift to using emerging internet-enabled technologies, including smartphones and wearable devices, for monitoring symptoms and behaviour, commonly referred to as either remote monitoring or ambulatory assessment^10^. This can be done using active symptom monitoring (ASM), for example reporting symptoms using a smartphone app, or through collecting passive sensor data such as physical activity levels and sleep data. These data could be used to spot early signs of relapse and provide opportunities for earlier intervention from services^11,12^.

The types of sensors used for passive data collection in smartphones and wearable devices include accelerometers, GPS sensors, environmental light and sound sensors, and photoplethysmogram (PPG) sensors. The raw data collected through these sensors can then be processed into features or variables, for example, distance travelled, resting heart rate, sleep duration or amount of sedentary behaviour. These features can then be grouped into different behaviours, for example, mobility, physical activity or sociability. This hierarchical framework is described in detail by Mohr et al.^13^. A glossary of technical terms can be found in the supplementary materials.

Collecting data from people continuously via technology produces huge volumes of data that can be used to identify an individual’s usual behavioural patterns. However, there are challenges when working with these data. First, if people do not carry or wear devices consistently, there may be a large amount of missing data and the accuracy and quality of the data collected through the devices are limited^14,15^. Second, reproducibility is a concern if researchers do not have access to the raw data and rely on the devices’ proprietary algorithms to generate features for analysis^14,16^. Finally, the amount of data can be challenging to analyse, and it is important to use methods that are appropriate for longitudinal data^17^.

A 2020 systematic review by Benoit et al.^18^ identified 51 studies using digital phenotyping in psychosis, focussing on describing the machine learning techniques used for analysis in 16 of those studies. Another review by De Angel et al.^19^ assessed digital monitoring tools in depression and identified key features associated with depression. Both reviews reported a large variation in the types of data and analysis methods used and highlighted inconsistencies in the reporting of methodology and the handling of missing data. However, neither of these reviews examined the methods of pre-processing raw passive data nor how features and behaviours were derived. There are currently no guidelines for reporting or any comprehensive summaries of the methods available for pre-processing and analysis for studies using passive data. Given the complexities and challenges of working with this type of data and the increasing use of emerging technologies^12^ a review of existing studies would be beneficial to researchers and aid in improving consistency and reproducibility of studies using passive data.

Therefore, this systematic review aimed to summarise how previous studies have collected, processed and analysed passive data collected through smartphones, wearable devices or research-grade devices to infer symptoms and other health-related information from people with psychosis or schizophrenia. Specific research questions were: (i) what sensors have been used in studies utilising digital data in psychosis or schizophrenia and what features have been derived?; (ii) how were the features derived from raw sensor data?; (iii) did the studies set thresholds for the amount of usable data for analysis and how were these defined?; (iv) what statistical methods have been used to analyse the data collected?

## Results

### Screening

**Fig. 1:**
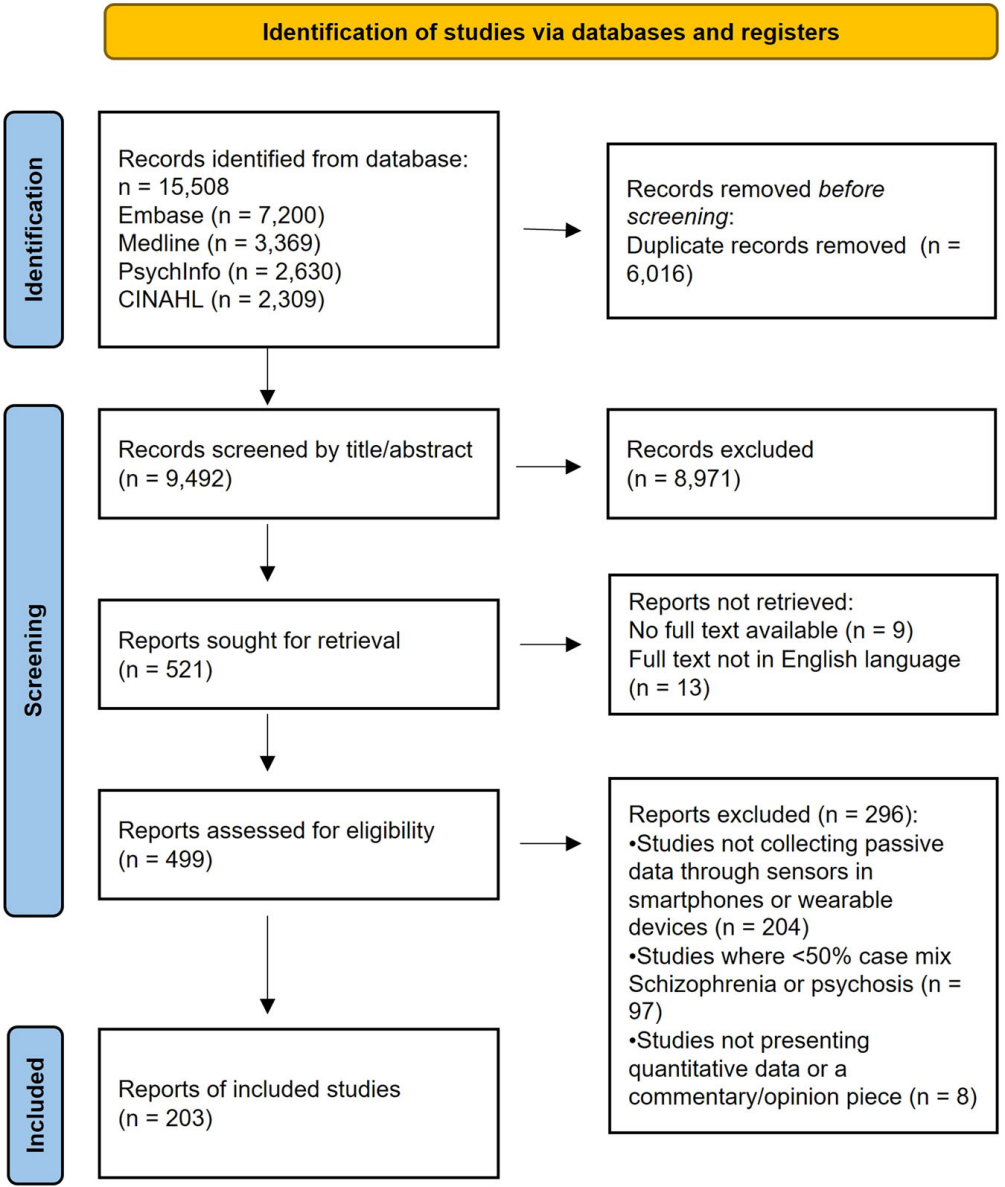
PRISMA flow diagram presenting the article screening process. The flowchart shows the number of articles identified by the searches across the four databases and the number removed at the title and abstract screening and full text screening stages. Of the 9492 unique articles identified in the searches there were 203 included in the review. The template for the diagram was taken from the PRISMA 2020 guidelines^100^.

Figure 1 displays the PRISMA flow diagram, including the reasons for exclusion at the full paper screening stage. A total of 15,508 records were identified through searching the four databases, including 6016 duplicates which were removed. After screening titles and abstracts of 9942 records, 8971 were excluded, with 521 remaining. Of those, there were 22 whose full reports were not available or not written in the English language; therefore, 499 had their full text screened for eligibility against the inclusion and exclusion criteria. There were 296 studies excluded at this stage, leaving 203 papers to be included in the review. The full list of papers included in the review can be found in Supplementary Table 3.

### Study characteristics

Table 1 summarises the characteristics of all included studies. The median total sample size for all the studies was 60 (IQR 29–100) and the median for the schizophrenia or psychosis sample only was 36 (IQR 20–66). There were 73 studies (36.0%) that included a healthy or population-based control group and 47 studies (23.2%) that included a mixed severe mental illness (SMI) clinical sample. For those mixed sample studies, the median percentage of the sample with schizophrenia or psychosis was 57.6% (IQR 48.8–74.1%) and the most frequently included other diagnoses were bipolar disorder (*n* = 30), depressive disorders (*n* = 26), mood disorders (*n* = 6), anxiety disorders (*n* = 6) and personality disorders (*n* = 4). The median age of participants across all studies was 40 years (IQR 35–46) and the median percentage of females was 40.0% (IQR 27.8–50.0%) (*n* = 19 did not report any demographic characteristics). Only 74 studies reported the ethnicity of the participants. The median years of illness duration was 14.0 (IQR 9.7–18.0, *n* = 64) and the median age at onset was 24.4 years (IQR 23.6–24.6, *n* = 13).

**Table 1:** Characteristics of the samples in the included studies

Characteristic	
Mixed clinical sample, *n* (%)	
Yes	47 (23.2%)
No	156 (76.8%)
% of mixed clinical samples with psychosis or schizophrenia, median (IQR)	57.6% (48.8–74.1%)
Sample size, median (IQR)	
Total	60 (29–100)
Psychosis or schizophrenia only	36 (20–66)
Control group, *n* (%)	
Yes	73 (36.0%)
No	130 (64.0%)
Control sample size, median (IQR)	34 (24–60)
Age, median years (IQR)^a^	40 (35–46)
Sex, median % (IQR)	
% female^b^	40.0% (27.8–50.0%)
% male^b^	59.9% (50.0–72.0%)
% other/transgender/non-binary^c^	3.10% (1.70–6.30%)
Clinical history, median (IQR)	
Illness duration^d^	14.0 (9.7–18.0)
Age at onset^e^	24.4 (23.6–24.6)
Ethnicity, median % (IQR)	
White or Caucasian^f^	47.9% (35.0–64.4%)
Black or African American^g^	32.9% (20.1–56.4%)
Multi-racial, bi-racial or mixed race^h^	6.0% (4.0–11.7%)
Asian or Asian-American^i^	5.0% (2.8–13.5%)

Reported in 183/203 studies^a^, 184/203 studies^b^, 8/203 studies^c^, 64/203 studies^d^, 13/203 studies^e^, 70/203 studies^f^, 52/203 studies^g^, 31/203 studies^h^, 24/203 studies^i^.

Over half of the studies (*n* = 111, 55.0%) collected passive data for less than 7 days, 39 studies (19.3%) collected for between 8 and 28 days, 50 studies (24.8%) collected for between 29 and 265 days and two studies collected data for over 1 year. Most studies only collected data from a single period (*n* = 179, 88.2%), with the remaining studies repeating passive data collection multiple times. For example, Gomes et al.^20^ collected 7 days of passive data at baseline which was then repeated at the end of a 16-week follow-up period.

Of the 203 studies included in the review, 123 reported details of how frequently data had been sampled and the duration of each sample (e.g. Torous et al.^21^ collected data for 1 min, every 10 min). Most of these studies that were collecting data through accelerometers specified the epoch duration for movement counts which ranged from 1 s^22^ to 2 min^23^. For studies collecting GPS data, the sampling frequency was up to 30 min when stationary and as little as every 10 s when moving^24^. Three studies^25–27^ collected GPS coordinates every 10 min or when the individual moved more than 10 m. Collection of audio data also varied, with sampling schedules ranging from every 2 min to 90 min.

### Devices and sensors

In 185 studies (91.1%), a single device was used for passive data collection, 16 studies (7.9%) used 2 devices and there were 2 studies where 3 devices were used. The most frequently used type of devices were research-grade accelerometers (*n* = 115, 51.6%), followed by smartphones (*n* = 46, 20.6%), smartwatches or commercial fitness bands (*n* = 21, 9.4%), and pedometers (*n* = 12, 5.4%). Most devices only used 1 sensor for data collection (*n* = 136, 62.1%), the maximum number used on a single device was 6 (*n* = 2, 0.9%) and there were four studies that did not specify what type of sensors the devices used.

**Fig. 2:**
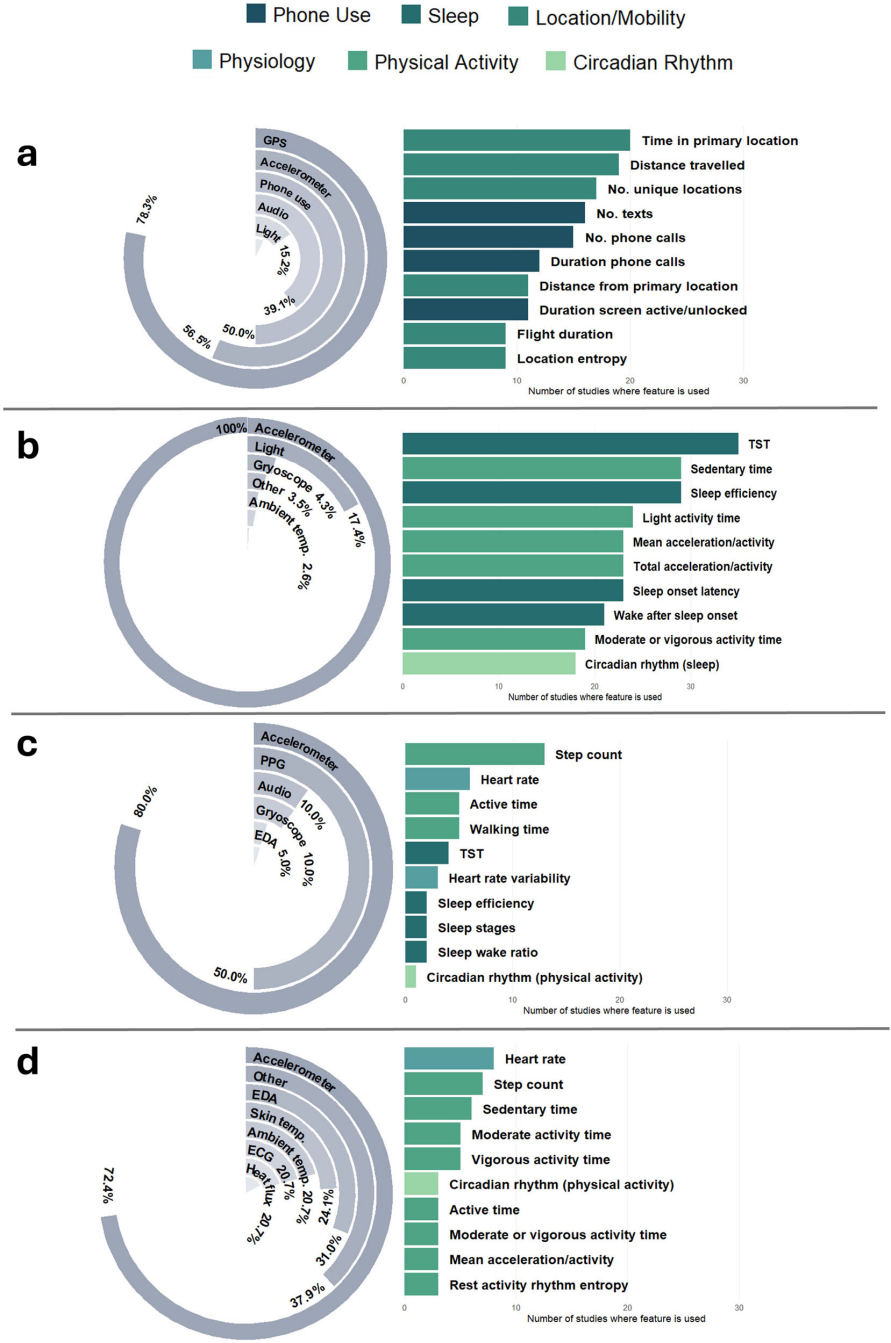
Sensors used by devices and features derived from sensor data for each type of device. The circular bar plots show the types of sensors used by each device group, with the bar representing the percentage of devices in each group using that sensor. The horizontal bar plots show the number of studies that used each feature, coloured by behaviour type (phone use, sleep, location/mobility, physiology, physical activity, and circadian rhythm). Figure **a** shows results for the smartphone device group, **b** for accelerometers, **c** for smartwatches & commercial fitness bands and **d** for other wearables. Findings for the pedometer device group are not shown. TST total sleep time, EDA electrodermal activity, ECG electrocardiogram.

Figure 2 shows the different sensors used by smartphones, smartwatches or commercial fitness bands, accelerometers and other wearables. Overall, the accelerometer was the most frequently utilised sensor, being used by 80.9% of devices (*n* = 178). Accelerometer sensors were the most frequently used in research-grade accelerometer devices, smartwatches commercial fitness bands, and other wearables. For smartphones, the most frequently used sensors were GPS (78.3%, *n* = 36), accelerometer (56.5%, *n* = 26) and phone use (47.7%, *n* = 21).

### Features and behaviours

There were 65 features derived from sensor data that were used in at least two different studies, of which the top five most commonly derived were sleep duration or total sleep time (*n* = 50 studies), time spent still or sedentary (*n* = 41), step count (*n* = 41), sleep efficiency (*n* = 32) and mean acceleration or activity count (*n* = 31). Figure 2 displays the top 10 features used for smartphones, smartwatches or commercial fitness bands, accelerometers and other wearables. For smartphones, the top three most common features were all in the location and mobility category, with time spent in primary location, distance travelled and number of unique locations being used 20 (43.5% of studies using a smartphone), 19 (41.3%) and 17 (37.0%) times, respectively.

Grouping these features by behaviour type the most frequently observed was physical activity, with 19 features, followed by sleep (13 features), phone use (10 features) and location or mobility (10 features). Physical activity and sleep features were the most frequent in studies using accelerometers, whilst for the smartwatches and other wearable devices physical activity and physiological features (e.g. heart rate) were common. (see Supplementary Tables 5 and 6 for a full list of features and behaviours).

### Data quality and quantity assessment

Ten studies reported technical issues with the devices, including defective or damaged devices^28–30^, malfunctioning devices^31^ or software^32^, syncing issues^33^, compatibility problems^34^ or unspecified technical problems^35–37^. The proportion of participants in the studies excluded for these reasons ranged from 0.5%^29^ to 14.8%^37^ (one study did not report the number affected^33^). Where studies used statistical methods (e.g. imputation) to handle missing data these are discussed in the “Analysis methods” section.

Thirty studies assessed data quality and excluded data in a variety of different ways. These are summarised in Table 2. Nine studies utilising actigraphs for data collection either visually inspected the data for errors or missingness, or compared the data to a written sleep log and assessed inconsistencies. There were five studies that assessed the quality of GPS data, including removing coordinates whose accuracy was below a certain threshold (ranging from 100 feet^38^ to 50 m^39^) and excluding participants whose travelling distance was greater than a threshold (e.g. travelled >80 miles per day^40^). Studies using physiological data identified outliers based on what is physiologically feasible; for example, skin temperature values < 20 or >40 °C^25^, skin conductance values < 0.1 or >39.95 µS^25^, and heart rate values <20 or >160 beats per minute (BPM)^41–43^. Outliers in accelerometer data were identified through visual inspection^44^ and applying other criteria, including values >30 m/s^2^^45^ or outside the range of the mean ± 3 standard deviations^46^. Of the 30 studies that assessed data quality, there were 13 (43.3%) reported how much data had been excluded or retained.

**Table 2:** Data quality assessments used in thirty studies and the amount of data removed or available for analysis

Data Type	Assessment	Amount of data removed/available
Actigraph	Compared to the written sleep log and identified inconsistencies	excluded 3 nights from *n* = 1^105^; NR^106,107^
	Visually inspected data for inconsistencies, missing data or artefacts	excluded 5–12% of data^88^; NR^108–111^
	Excluded participants with invalid recordings	excluded 6.8% of data^112^
Audio	Excluded inaudible files	excluded 28 files (3.7%)^113^; NR^114^
	Excluded corrupt recordings	NR^115^
GPS	Excluded samples with estimated accuracy over the specified threshold:	
	35 m	excluded 5% of samples^27^; NR^25^
	100 feet	retained a mean of 1320.4 accurate samples^38^
	50 m	NR^39^
	Excluded outliers:	
	values with change in coordinates >200 km/h	NR^27^
	values indicating distance from home >400,000 m	excluded 3% of samples^27^
	travelled >80 miles per day or 4 SDs above the mean	excluded *n* = 3(1.7%)^40^
	median distance > 4 SDs above the mean	excluded *n* = 2 (1.3%)^38^
Physiological data	Excluded outliers/artefacts in HRV data:	
	RR intervals >2000 ms and <300 ms	excluded *n* = 14 (58.3%)^73^
	visually inspected data	corrected artefacts using software algorithm^44^
	Excluded outliers/artefacts in HR data:	
	values < 20 BPM	NR^41^
	values < 20 BPM and >160 BPM	NR^42,116^
	insufficient sampling rate (>15 min or >10 min between values)	NR^41,42^
	Excluded outliers/artefacts in EDA data:	
	values < 0.1 or >39.95 µS	NR^25^
	Excluded outliers/artefacts in skin temperature data:	
	values < 20 or >40 °C	NR^25^
Accelerometer data	Excluded poorly calibrated data	excluded *n* = 11 (0.01%)^117^; excluded <0.1% of participants^118^ ; NR^47^
	Excluded outliers/artefacts:	
	values > 30 m/s^2^ and >2 times the IQR	excluded 0.1–8% of data^45^
	values outside mean ± 3 SDs	excluded *n* = 9 (3.6%)^46^
	post-calibration error >0.02 g	NR^57^
	visually inspected data for artefacts and missingness	excluded *n* = 8 (5.6%)^44^
	Excluded intervals with <2 h of missing data	NR^74^

There were 69 studies that applied a threshold of data quantity, either to periods of data collected or to study participants. Eight of these studies did not specify the exact threshold used. For example, Wulff et al.^23^ stated days with missing data of several hours were excluded and Dennison et al.^47^ excluded individuals with insufficient device wear time; however, neither defined their thresholds explicitly. Table 3 summarises the thresholds applied in the remaining 61 studies. There were 26 studies, all using accelerometers, that defined both the number of hours of data for a day to be considered “valid” and the number of valid days within the study period needed for a participant to be included in the analysis. The threshold for a valid day ranged from 6 h to 16 h of data, and the number of minimum valid days ranged from 2 to 10 days.

**Table 3:** Data quantity thresholds applied and the amount of data removed or available for analysis

Threshold type	Threshold	Amount of data removed/available
Defining valid days and the number of valid days	≥6 h per day for ≥3 days	Excluded *n* = 19 (9.2%)^119^; *n* = 3 (2.4%)^29,30^; *n* = 24 (32.4%)^120^; *n* = 19 (9.1%)^121^; NR^81^
	≥8 h per day for ≥10 days	Excluded *n* = 2 (10.5%)^20^; NR^122^
	≥10 h per day for ≥2 days	NR^123–126^
	≥10 h per day for ≥3 days	Excluded *n* = 2 (3.6%)^127,128^; *n* = 17 (31.5%)^129^; *n* = 10 (8.8%)^130^
	≥10 h per day for ≥4 days	Excluded *n* = 2 (6.7%)^131^; *n* = 7 (14.6%)^132^; *n* = 11 (16.7%)^133^ ; NR^56,134–138^
	≥10 h per day for ≥5 days	Excluded *n* = 38 (14.7%)^139^; NR^140^
	≥16 h per day for ≥4 days	76% valid data at baseline, 52% at 12 months^57^
	≥19 h per day for ≥7 days	NR^50^
	≥1368 min per day for 7 days	Excluded *n* = 13 (9.2%)^36^; NR^141^
	<10% missing data per day for ≥7 days	Excluded *n* = 4,345 (4.2%)^117^
	data in each hour of 24 h day for ≥6 days	Excluded *n* = 6,020 (5.8%)^117^
	<1 h non-wear per night for 4 nights	Excluded *n* = 2 (6.7%)^131^
Defining valid days only	≥19 h per day	NR^24,48,49^
	≥20 h per day	NR^73^
	≥21 h per day	NR^75,111^
	≥ 23 h per day	NR^142^
	24 h	Excluded *n* = 3 (4.8%)^143^
Defining the number of days/nights only	≥3 days	Excluded *n* = 11 (4.4%)^46^; *n* = 2 (8.3%)^144^; *n* = 1 (2.5%) at baseline & *n* = 19 (47.5%) at 6 months^145^; NR^106^
	≥3 nights	Excluded *n* = 10 (5.0%)^28^; NR^80^
	≥5 days	NR^74^
	≥7 days	Excluded *n* = 2 (11.8%)^51^
	≥10 days	Excluded *n* = 2 (3.2%)^52^
Using data values	≥300 steps recorded	NR^31,53^
	>0 steps recorded	NR^54^
Other	total wear time ≥72 h	*n* = 6,978 (6.7%)^118^
	average on-body time ≥1368 min per day	*n* = 4 (3.4%)^146^; NR^147,148^
	continuous recordings of ≥8	NR^149^
	>10 missing endpoints (derived from either passive data or imaging)	*n* = 16 (21.3%)^77^

Six studies using data collected through smartphones in the CrossCheck study^48^ applied thresholds. Four of these studies^24,48–50^ applied a quantity threshold of 19 hours of data for a day to be included, whilst the other two studies specified thresholds of either 7^51^ or 10 days^52^ of data for participants to be included in analysis. There were three studies that used Fitbit devices to collect data and used step count to define valid days. Two of these used a threshold of 300 steps^31,53^ whilst another removed days from analysis where zero^54^ steps had been recorded.

Seventeen studies specified how non-wear time had been defined, with the majority using actigraphs (15/16). Twelve of these used an activity count of zero for a specified timeframe (between 10 and 90 minutes depending on the study) to identify non-wear time periods. Two studies stated non-wear had been flagged by device software^55^ or an analysis package^56^ and another identified non-wear as periods where the standard deviation in 2 out of the 3 axes was less than 13 mg, or the value range is less than 50 mg^57^. Algorithms developed by Troiano et al.^58^ and Choi et al.^59^ were cited as methods used for defining non-wear time in actigraphy data. The study by Martanto et al.^60^ collecting data through Fitbits used a lack of heart rate data to indicate non-wear time.

Finally, four studies collecting data through smartphones defined data quality as the quantity of recorded data as a proportion of the expected quantity according to the sampling frequency. Cohen et al.^61^ reported overall average passive data quality of 57.4%, Lakhtakia et al.^62^ reported mean GPS data quality of over 50% at each of their study sites and Henson et al.^63^ reported collection rates of 72% and 60% for GPS and accelerometer data, respectively.

### Data processing methods

For processing the raw data, there were 39 actigraphy studies that stated the software and version used. ActiLife (Actigraph) was the most frequently used software (n = 19), followed by Actiwatch (Cambridge Neurotechnology, n = 13) and Actiware (Philips, n = 7). For studies using other wearables, there were three studies that explicitly stated they had used the Fitbit algorithms to derive features, five that had used the SenseWear software and one that had used the Empatica algorithms. Some studies collecting data through smartphones referred to both the Google Activity Recognition API (n = 4) and Android proprietary algorithms for location and activity features (n = 3).

Several other methods were cited as being used, including papers by Barnett et al.^64^ for deriving location and mobility features from GPS data with missingness, Brond et al.^65^ and Bai et al.^66^ for processing accelerometer data, and Menghini et al.^67^ and Cole et al.^56,68^ for sleep features. The DBSCAN and Haversine algorithms were both used multiple times for location clustering and calculating the distance between locations, respectively. Open-source software was also utilised, including Kubios and Ledalab software for analysing HRV data, the Cortex platform for processing smartphone data and DPSleep^69^ for sleep data. Three R packages were cited, which were rVAD^70^ for voice detection, GGIR^56,71^ for calculating sleep and activity features and the nparACT^72^ package for deriving circadian rhythm features.

### Analysis methods

As well as applying thresholds for data usability, as described above, there were several additional approaches used to handle missing data. Eleven studies imputed missing data, replacing the missing values with either the average value^23,52,73–77^ for the recording period or the nearest value that was recorded^52,53,78,79^. For example, one study recorded environmental light using an actigraphy, and if a 2-minute epoch was missing a light value then it was substituted with the value from the closest recorded epoch^79^. Other methods used to impute missing data were a regularised iterative principal components algorithm^80^ and multiple imputation by chained equations (MICE)^81^, where the missing value is estimated multiple times by a model using the available data. Three studies^24,82,83^ used the amount of missingness as a feature in their analysis (e.g. number of days or minutes of missing data). Liebenthal et al.^82^ found a significant association between the number of days where phone data was missing ( < 60 mins recorded) and the PANSS^84^ P2 domain (conceptual disorganisation), but the other two^24,83^ did not report results specific to the missingness features. An additional study used a logistic regression model with missing data as the outcome and baseline variables as possible predictors of missingness, then used any variables found to be predictive in their mixed-effects model^85^. Reinertsen et al.^42^ applied an algorithm classifying participants with schizophrenia from healthy controls using contiguous sliding windows of daily heart rate and accelerometer data. If the data for a given day was missing ( < 50 data points), no features were derived, and no prediction was made for that day. The 2020 study by Adler et al.^52^ categorised missing data as either type 1 (where data from one sensor was missing but other data had been collected) or type 2 (where data from all sensors was missing for the same period). Type 1 missing data was replaced with zeroes whilst type 2 data was substituted with the average for that hour, unless it was location data whereby the last recorded location was used. Only three studies^52^^,63,86^ reported the amount of data that was missing, which ranged from 19%^86^ to 72%^63^.

Most of the reviewed studies (n = 142) either did not specify how they had utilised the repeated measurements or had calculated a single value (sum or average) of the features across the whole of their data collection period. For studies that utilised longitudinal information, the most frequently used methods were mixed effects models, where random effects were used to distinguish within-person and between-person variation. In the 21 studies that used mixed effects models some used daily summaries of variables in their model, whilst others divided up the follow-up period into multiple time windows and aggregated features over these windows. For example, Kalisperakis et al.^78^ initially calculated daily features and then used monthly summaries (mean and standard deviation) of those features in the mixed effects models. There were eight studies that used time series modelling methods, including generalised estimating equations^87^, periodogram analysis^23^, partial autocorrelation functions^88^, graph/network algorithms^43,76^ and anomaly detection methods^51,61^. Other studies used more comprehensive machine learning techniques which can be applied to more granular data than other methods such as mixed effects models. Examples include neural networks^83,89,90^, support vector machines^41,42^ and an unsupervised clustering algorithm^91^.

### Study aims and outcomes

Most of the studies (116 out of 203) included in the review assessed the correlation or association between passive data and either clinical assessment scores, diagnostic status, medication adherence or active symptom monitoring data. There were 39 studies using passive monitoring to assess the effectiveness of a medication or other intervention (e.g. an exercise programme intervention), 16 developing or validating methods, tools or devices for use in passive monitoring, and 15 assessing the feasibility or acceptability of an intervention and/or of passive monitoring.

There were 20 studies that developed models either predicting clinical outcomes or classifying participant groups; these are summarised in Table 4 and discussed further in the following section. Seven of these studies aimed to distinguish people with schizophrenia from healthy controls or from people with other serious mental illnesses including mood disorder and major depressive disorder. Five studies used passive data to predict EMA/ASM scores and there were six studies aiming to identify or predict behaviour anomalies in the period near to a relapse. Two studies developed models to predict the risk of relapse within the following week. An additional study^92^ initially aimed to identify signatures of relapse, however, there were not enough occurrences of relapse during their study period so instead assessed the feasibility of passive data collection.

**Table 4:** Recommendations for reporting in studies using passive data, based on the findings from this review

Study and participants	How many participants were in the study? How long was passive data collected for? How many participants who entered the study did not complete the full period of passive data collection? Is this study using newly collected data or using an existing dataset? (e.g. CrossCheck)
Devices	What devices were used, including manufacturer and model? What instructions were given to participants about wear? (e.g. location of device, any removal periods) What sensors did the devices use to collect data? What device settings were selected? (e.g. epoch length, if applicable)
Data pre-processing	Was data extracted raw, or processed by device algorithm? What pre-processing was done? (e.g. removing outliers, splitting data into time windows, identifying and removing (or replacing) non-wear time) If all data recorded was not analysed, how was data sampled or selected for analysis?
Data quality	Was there any assessment of data quality? (e.g. whether sampling rate was as expected) Was a threshold of quantity of data applied? (e.g. a minimum of 10 h of data per day) If so, how much data was included/excluded for each participant?
Feature extraction	What specific features were extracted from raw data? Including their unit of measurement, data channels contributing to them, and the time window (e.g. number of steps per day, or number of outgoing phone calls per hour) How were these extracted from raw data? Were any open-source algorithms used?
Analysis methods	How was missing data handled? How were repeated observations utilised? (e.g. were they summarised over hours, days or weeks?) Was any feature or variable selection performed?

### Prognostic factor and prognostic model studies

The majority of prognostic studies collected data through smartphones (12 out of 20), with the others using smartwatches *n* = 2), actigraphy devices (*n* = 4) and wearable adhesive patches (*n* = 3). All seven studies aiming to classify people with psychosis or schizophrenia from other groups used physical activity metrics amongst others. For the 12 studies collecting data through smartphones, the most frequent behaviours used were location mobility and phone use, whilst for other devices the most frequent behaviours were physical activity, physiology and sleep. Most used machine learning methods for their analysis, including random forests, anomaly detection algorithms, neural networks and support vector machines. Non-machine learning methods included generalised estimating equations and mixed effects regression. Where appropriate the risk of bias for each study was assessed using either the PROBAST or QUIPS tools. Most studies were deemed to have a moderate or high risk of bias in relation to the measurement of confounders and description of the study sample. The key findings from each study are displayed in Supplementary Table 7, and the quality assessment can be found in Supplementary Tables 8 and 9.

## Discussion

The use of smartphones for passive data collection is increasing, with smartwatches and commercial bands less commonly used (see Supplementary Fig. 1). Research-grade accelerometers, including actigraphy devices, were the most frequently used devices. The most frequently measured features were those related to physical activity and sleep, however, this did vary by device type. Most of the studies did not report if they had assessed the data quality or applied a data quantity threshold, and of those that did there were only a handful that reported the amount of data that was excluded or available for analysis. Some studies, particularly those using actigraphy devices, relied on the features derived by the device algorithms whilst others did not state whether they had access to raw data or did not sufficiently describe the methods used to pre-process the data.

Few studies reported the amount of missing data or whether statistical methods had been used (e.g. imputation) to handle missingness. For passive data, the level of missingness can be significant due to potential technical issues with devices transferring of data or participants not wearing devices consistently. Whilst some studies did apply thresholds based on the quantity of data collected the disadvantage of this is that it could lead to a reduction in sample size and removal of some data that still could be useful despite high levels of missingness. There was also little justification for why certain thresholds (e.g. 10 h of data per day) had been chosen. Some studies had used methods to impute missing data (e.g. imputing by average or multiple imputation) that are commonly used in statistical modelling, however it is not clear whether these methods are suitable for this type of high frequency data and there is currently no guidance for researchers on what is the best approach to use. A method developed by Barnett et al.^64^, which was used in a few studies in the review, derives features from GPS data where missingness is present. Given the variation in types of data collected through passive sensors, it may be that separate methods are needed for each type. However, this adds an additional complexity to the processing of the data. Another approach used in three studies was to use the amount of missing data as a feature in itself. The study by Liebenthal et al.^82^ reported an association between the number of days of missing phone data and PANSS scores for conceptual disorganisation, indicating that the absence or presence of passive data could potentially be used as a marker of an individual’s health.

Most studies used a single value (e.g. mean or total) to summarise repeated observations over the follow-up period, therefore, not utilising the amount of data to its potential and not capturing the longitudinal patterns of behaviour. Generalised linear mixed-effects models (GLMMs) were used to account for repeated measurements in a few studies and machine learning methods are being increasingly used. GLMMs can model the variation in observations within each individual and between individuals, utilising more of the data collected to model a pattern of behaviour. However, the studies using these models still summarised variables over a period (either daily or weekly). Whilst machine learning methods, such as neural networks and LSTMs, are capable of utilising the granular, high-frequency data, they do usually require large sample sizes for training the models to avoid overfitting (where a model is not generalisable and underperforms when applied to new data). Most of the studies in this review were in relatively small samples (median 60). There is guidance for sample size calculations when developing clinical prediction models, however, there are not any specifically for machine learning algorithms. The other potential drawback of using machine learning models is they can be harder to interpret and explain. This is important for researchers who are interested in identifying the specific passive features or behaviours that are associated with an outcome, e.g. relapse. Some machine learning models (e.g. random forests) are more explainable than others and there are methods to evaluate the importance of features in the models.

The lack of reporting on methodology found in the articles included in this review is consistent with similar reviews by Benoit et al.^18^, De Angel et al.^19^ and Rohani et al.^93^. There were some open-source methods and software used in the studies, for example the GGIR package, but few instances of researchers making their analysis code available. The inconsistencies in how passive data is collected and processed make it difficult to compare findings from any studies or reproduce them.

There are some limitations to our review. Due to the differences in study design in the included articles we did not assess the quality of all studies, only those that were assessing potential prognostic factors or developing prediction models. As there was significant variation in the passive data features used, outcomes assessed, and modelling strategies used we did not attempt to perform meta-analysis with any of the studies. We included studies that collected passive data from people with psychosis and schizophrenia. Passive data collection through wearable devices and smartphone sensors are being used in a range of clinical areas, such as cardiovascular disease^94^ and rheumatoid arthritis^95^. By limiting our search to mental health, we may have missed some studies that are using relevant methods. Similarly, there may be newer methods proposed that have not yet been applied to any particular clinical setting and will also have been missed. We also did not check whether included studies had published or pre-registered their protocols, which may have included more detail of their data processing and analysis plans.

Some of the articles included in the review were using data collected in the same study, for example, the data from the CrossCheck^48^ project was used in at least 9 studies and there were 3 studies using an open source dataset called Psykose^96^. They were included as separate studies in the review as they did not necessarily use the same methods or the same features in their analyses. This does mean, however, that our estimates of how frequently some devices are being used in these types of study are overestimated. Some of the studies using the CrossCheck data appeared to be using the raw data, whilst others were using the freely available pre-processed version consisting of hourly and daily summaries. Whilst the publishing of open-source datasets is, in general, a positive step forward in terms of open science, it is still important that researchers using this data are provided with sufficient information about how the data were pre-processed. It is also important that the methods in studies that have re-used data make it clear whether or not the raw data has been used, whether any additional exclusion criteria have been applied, or if there has been any additional processing, feature extraction or feature selection performed. Additionally, it should be clearly stated which other published papers have used the same dataset.

In order to increase the reproducibility and validity of studies in this area of research there is a need to significantly improve the reporting of methodologies used, including specifics about the type and models of devices used, the pre-processing and any quality assessment of data, and the handling of missing data. Additionally, to improve the consistency of passive data analysis the availability and use of open-source or standardised methods should be encouraged. This is particularly important given the range of devices currently available for remote monitoring and the emergence of new technologies, such as smart rings^97^. These devices and the data they collect could enable remote monitoring across a range of health conditions. However, for this to be successful there do need to be improvements in the way this data is currently being used. As well as the methodologies, other aspects of these types of study, for example, the occurrence of adverse events, have also been found to be under-reported^98,99^. Although creating full guidelines for standardised analysis and reporting of passive sensing data would require input from experts from across the field (e.g. through a Delphi study) and is outside the scope of this paper, Table 4 provides a brief checklist of reporting recommendations based on the findings of the current systematic review.

To conclude, the collection of passive data from wearable devices of people with psychosis and schizophrenia is increasing. However, the reporting of methods used to process and analyse the data is inconsistent, making reproducible research difficult. There is a need, therefore, to improve the standardisation of methods and reporting in this area of research.

## Methods

The review protocol was registered prospectively on PROSPERO (CRD 469868). Reporting for the review is in line with the Preferred Reporting Items for Systematic Reviews and Meta-Analyses (PRISMA) statement^100^ (see Supplementary Table 4 for the completed checklist).

### Literature search and study selection

Four databases (Embase, PsychInfo, Medline through Ovid and CINAHL through EBSCO) were searched to identify articles for inclusion in the review. The searches were first performed on 13th June 2023 and then updated on 24th April 2024. The search strategy combined terms relating to psychosis and schizophrenia with terms either relating to smartphones and wearable devices or with actigraphy and accelerometers. The search containing terms relating to smartphones and wearables was limited to articles published from 2007 onwards, as that is the point when smartphones and commercial wearables were available. The search containing terms related to accelerometers and actigraphs was not limited by year of publication. The full search strategy, including the exact terms used, can be seen in Supplementary Tables 1 and 2.

Inclusion criteria were published, peer-reviewed articles collecting any type of passive data through smartphones or wearable devices. We defined wearable devices as any device worn on any part of the body, including research-grade devices (such as accelerometers, pedometers and actigraphs) and commercial devices (such as smartwatches and fitness trackers). We defined passive data as data that had been collected for a period of at least 24 h in uncontrolled settings and when participants had not been given a specific activity to perform. For example, a study where participants in a laboratory wore an accelerometer for 1 h whilst performing specific tasks was not considered passive data. Included studies must have had more than, or equal to, 50% of participants with psychosis or schizophrenia in their clinical sample, or if lower than 50% must have reported results separately for the schizophrenia and psychosis group. Studies were excluded if they used smartphones to collect active symptom monitoring data only, had collected passive data using sensors in a non-wearable device, were not written in English, were non-empirical studies (literature reviews, commentary or opinion pieces, and editorials), were qualitative studies, were published as a pre-print only or if the full text could not be accessed.

All identified articles were exported to EndNote Desktop (version 20.4.1). Duplicates were removed, and all abstracts were screened by one reviewer (SBl) against the inclusion and exclusion criteria. The remaining full-text articles were screened by one reviewer (S.B.l.), with 20% second screened by one of three other reviewers (S. Bu, E.E. and S.F.), with disagreements discussed to reach a consensus (inter-rater reliability coefficient 0.81). The screening was managed using a Microsoft Excel proforma with reasons for inclusion and exclusion during the full article screening recorded.

### Data extraction and synthesis

A data extraction form was piloted with five studies before being revised by reviewers SBl, SBu, SF and EE. Data extraction for each study was carried out by either SBl, SF, EE or AO. Data extracted for each study included: (i) study sample characteristics, (ii) data collection, including types of devices and sensors utilised, (iii) features and behaviours derived from the passive data, (iv) methods used to pre-process and analyse the data, including whether data quality was assessed, (v) outcome of interest in the study and (vi) analysis methods used, including methods to handle missing data and repeated/longitudinal measurements.

Following data extraction, devices were categorised into the following groups: (i) smartphones; (ii) smartwatches and commercial fitness bands; (iii) research-grade accelerometers (including actigraphy devices); (iv) pedometers; and (v) other wearables (including research-grade wrist/arm bands, heart rate monitors and adhesive patch sensors). For each device category, the types of sensors used were summarised (e.g. GPS sensor), as well as the features or variables derived from the raw sensor data and the behaviours that can be inferred from the features. Features were grouped into the following behaviour types (i) physical activity, (ii) sleep, (iii) phone use^13^, (iv) location or mobility, (v) environment, (vi) sociability, (vii) physiology, and (viii) circadian rhythm.

### Quality assessment

For longitudinal observational studies conducting prediction^101^ or prognostic factor^102^ or predictive modelling, additional information was extracted, including methods of variable selection, modelling and validation. These studies were also assessed for quality using either the Quality in Prognosis Tools (QUIPS)^103^ or the Prediction model Risk Of Bias Assessment Tool (PROBAST)^104^ for prognostic factor and prediction model studies, respectively.

## Supplementary information

Supplementary Information

## Data Availability

The datasets used and/or analysed during the current study are available from the corresponding author upon reasonable request.
