# Supplementary material for: A systematic review of passive data for remote monitoring in psychosis and schizophrenia
Source: NPJ Digit Med. 2025 Jan 27;8:62. doi: 10.1038/s41746-025-01451-2 (PMC11772847; doi:10.1038/s41746-025-01451-2)
Supplement: Supplementary file 1 — Supplementary Information [file 41746_2025_1451_MOESM1_ESM.pdf]

Supplementary file for *A systematic review of passive data for remote monitoring in psychosis and schizophrenia*

Glossary of terms

Epoch – duration of recording intervals. Term is commonly used in relation to accelerometer and actigraphy devices, where activity counts are summarised for a defined epoch, e.g. 30 seconds, 1 minute, 5 minutes etc.

Pedometer – device that measures an individual's movement and converts it into a step count. Can either contain a mechanical switch or accelerometer sensors that detect movement

Actigraphs – devices commonly used in research settings to monitor people's rest/activity profiles. They use accelerometers to measure activity, some also contain gyroscope and light sensors.

Accelerometer - electronic sensor that measures the acceleration forces acting on an object, to determine the object's position in space and monitor the object's movement.

Magnetometer - a sensor that converts the magnitude and variations of a magnetic field into electric signals. Magnetic fields, as exemplified by the magnetic field of the earth (earth magnetism) or magnets are familiar yet invisible phenomena.

Gyroscope - device used for measuring or maintaining orientation and angular velocity.

Global Positioning System (GPS) Sensors - receivers with antennas that use a satellite-based navigation system with a network of 24 satellites in orbit around the earth to provide position, velocity, and timing information. Collects the relative degrees of latitude and longitude data from GPS and/or using cell tower and WiFi signals.

Photoplethysmography (PPG) - consists of a light source, typically an LED, and a photodetector. The LED emits light that is absorbed by the tissue in the wrist, and the photodetector measures the amount of light that is transmitted through or reflected from the tissue. PPG is a non-invasive technology that uses a light source and a photodetector at the surface of skin to measure the volumetric variations of blood circulation.

Microphone – is a sound sensor converting sound to electric signals.

Bluetooth - is a short-range wireless technology standard that is used for exchanging data between fixed and mobile devices over short distances. Devices connected in a Bluetooth network communicate with each other using ultra-high frequency (UHF) radio waves, which are electromagnetic waves.

Temperature sensor - physical sensor that detects and measures heat and cold, and converts it into an electrical signal.

Light sensor - an ambient light sensor is a component in smartphones, notebooks, other mobile devices, automotive displays and LCD TVs. It is a photodetector that is used to sense the amount of ambient light present, and appropriately dim the device's screen to match it.

Features – variables that are extracted or derived from raw sensor data. For example, raw accelerometer data can be transformed into activity counts, step counts or minutes of sedentary behaviour etc.

Digital phenotyping - “moment-by-moment quantification of the individual-level human phenotype in-situ using data from smartphones and other personal digital devices.” Torous et al, 2016, doi: 10.2196/mental.5165

Search strings

1. psychosis or psychoses or psychotic or schizo* or SMI or “severe mental illness” or “serious mental illness” or paranoia or paranoid or hallucinat* or delusion* or grandios* or suspicio* or “thought disorder” or “positive symptoms” or “negative symptoms” or “hearing voices” or “voice hear*” or “unusual beliefs” or persecut*

   AND either

2. "digital monitoring" or "digitally monitor" or "remote monitoring" or "remotely monitored" or "RMT" or "remote measurement technologies" or passive* or sensor or sensors or sensing or wearable or smartphone or "smart phone" or "mobile phone" or apps or app or "smart watch" or smartwatch or mhealth or "digital biomarker" or "digital phenotyp*" or “wrist-worn” or “electronic monitoring” or “GPS” or “Global Positioning System” or “remote sensing technology” or “nearables”

   OR

3. acceleromet* or pedomet* or actigraph* or “bed sensor” or “motor activity” or “psychomotor activity” or “rest-activity” or “rest activity” or “physical activity”

Amendments to protocol

The review was registered on Prospero (CRD 42023469868). Two amendments were made to the protocol after registration:

1. Additional exclusion criteria – studies where passive data was collected in controlled experimental conditions only, or only during certain tasks
2. Due to the number of studies included and the variation of types of studies we decided to only do quality assessment on studies where they had developed or validated a prediction model or assessed prognostic factors. We felt this was appropriate as the main focus of our review was to describe the methods used to process passive data, not the outcomes of the individual studies.

Database searches

Searches first run on 13th June 2023, then updated on 24th April 2024.

Supplementary Table 1. *Search strategy for Embase & Medline, both accessed through Ovid.*

Search	Terms
1	(psychosis or psychoses or psychotic or schizo* or SMI or “severe mental illness” or “serious mental illness” or paranoia or paranoid or hallucinat* or delusion* or grandios* or suspicio* or “thought disorder” or “positive symptoms” or “negative symptoms” or “hearing voices” or “voice hear*” or “unusual beliefs” or persecut*) ti,kw,ab
2	"digital monitoring" or "digitally monitor" or "remote monitoring" or "remotely monitored" or "RMT" or "remote measurement technologies" or passive* or sensor or sensors or sensing or wearable or smartphone or "smart phone" or "mobile phone" or apps or app or "smart watch" or smartwatch or mhealth or "digital biomarker" or "digital phenotyp*" or "wrist-worn" or "electric monitoring" or "GPS" or "global positioning system" or "remote sensing technology" or "nearables").ti,kw,ab
3	1 and 2
4	Limit 3 to 2007 onwards
5	(acceleromet* or pedomet* or actigraph* or "bed sensor" or "motor activity" or "psychomotor activity" or "rest-activity" or "rest activity" or "physical activity").ti,kw,ab.
6	1 and 5
7	4 or 6

Supplementary Table 2. *Search strategy for PsychInfo, accessed through Ovid.*

Search	Terms
1	(psychosis or psychoses or psychotic or schizo* or SMI or “severe mental illness” or “serious mental illness” or paranoia or paranoid or hallucinat* or delusion* or grandios* or suspicio* or “thought disorder” or “positive symptoms” or “negative symptoms” or “hearing voices” or “voice hear*” or “unusual beliefs” or persecut*) ti,,ab
2	"digital monitoring" or "digitally monitor" or "remote monitoring" or "remotely monitored" or "RMT" or "remote measurement technologies" or passive* or sensor or sensors or sensing or wearable or smartphone or "smart phone" or "mobile phone" or apps or app or "smart watch" or smartwatch or mhealth or "digital biomarker" or "digital phenotyp*" or "wrist-worn" or "electric monitoring" or "GPS" or "global positioning system" or "remote sensing technology" or "nearables").ti,ab
3	1 and 2
4	Limit 3 to 2007 onwards

5	(acceleromet* or pedomet* or actigraph* or "bed sensor" or "motor activity" or "psychomotor activity" or "rest-activity" or "rest activity" or "physical activity").ti,ab.
6	1 and 5
7	4 or 6

Search strategy for CINAHL, accessed through EBSCO

TX ((psychosis or psychoses or psychotic or schizo* or SMI or "severe mental illness" or "serious mental illness" or paranoia or paranoid or hallucinat* or delusion* or grandios* or suspicio* or "thought disorder" or "positive symptoms" or "negative symptoms" or "hearing voices" or "voice hear*" or "unusual beliefs" or persecut*)) AND TX (("digital monitoring" or "digitally monitor" or "remote monitoring" or "remotely monitored" or "RMT" or "remote measurement technologies" or passive* or sensor or sensors or sensing or wearable or smartphone or "smart phone" or "mobile phone" or apps or app or "smart watch" or smartwatch or mhealth or "digital biomarker" or "digital phenotyp*" or "wrist-worn" or "electric monitoring" or "GPS" or "global positioning system" or "remote sensing technology" or "nearables"))

TX ((acceleromet* or pedomet* or actigraph* or "bed sensor" or "motor activity" or "psychomotor activity" or "rest-activity" or "rest activity" or "physical activity")) AND ((psychosis or psychoses or psychotic or schizo* or SMI or "severe mental illness" or "serious mental illness" or paranoia or paranoid or hallucinat* or delusion* or grandios* or suspicio* or "thought disorder" or "positive symptoms" or "negative symptoms" or "hearing voices" or "voice hear*" or "unusual beliefs" or persecut*))

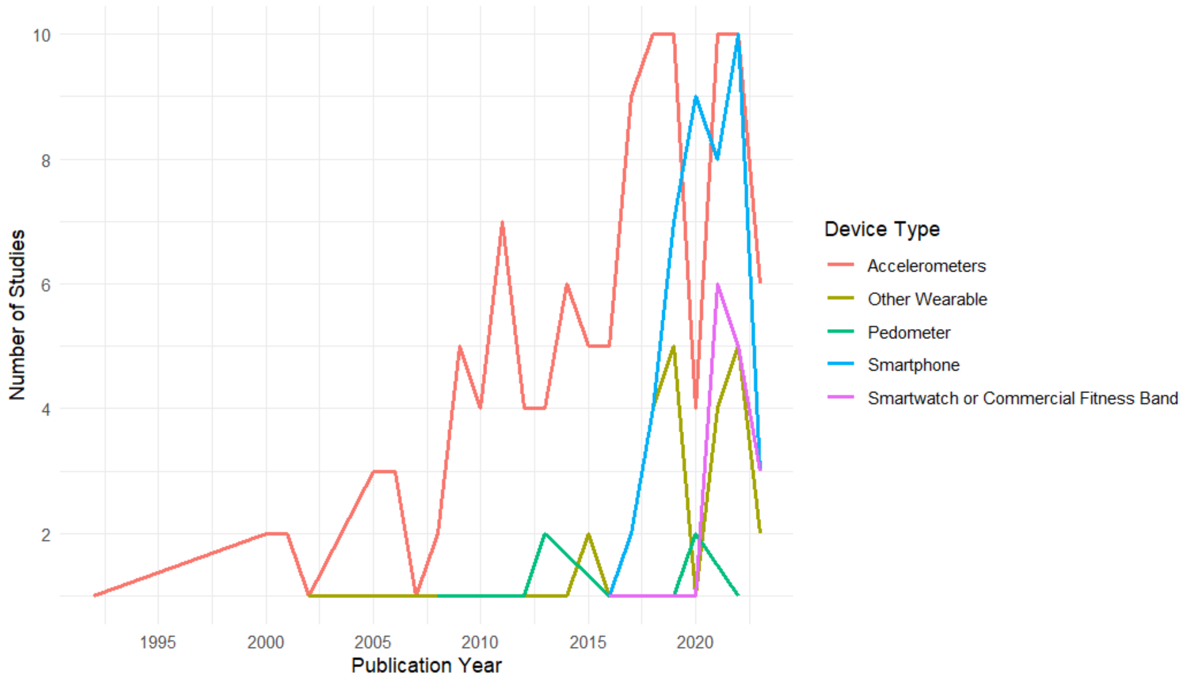

Supplementary Figure 1. *Device type by publication year for all included studies.* Each line represents a different device type.

Supplementary Table 3. *List of all studies included in the review.*

1st Author	Year	Title
Abel, D. B.	2021	Quality versus quantity: Determining real-world social functioning deficits in schizophrenia.
Abel, D. B.	2021	Social functioning in schizophrenia: Comparing laboratory-based assessment with real-world measures.
Abplanalp, S. J.	2021	Feasibility of using smartphones to capture speech during social interactions in schizophrenia.
Adler, D. A.	2020	Predicting Early Warning Signs of Psychotic Relapse From Passive Sensing Data: An Approach Using Encoder-Decoder Neural Networks.
Adler, D. A.	2022	Machine learning for passive mental health symptom prediction: Generalization across different longitudinal mobile sensing studies.
Afonso, P.	2010	Discrepant nocturnal melatonin levels in monozygotic twins discordant for schizophrenia and its impact on sleep.
Afonso, P.	2011	Schizophrenia patients with predominantly positive symptoms have more disturbed sleep-wake cycles measured by actigraphy.
Afonso, P.	2011	Sleep-promoting action of the endogenous melatonin in schizophrenia compared to healthy controls.
Afonso, P.	2014	Sleep-wake patterns in schizophrenia patients compared to healthy controls.
Amiri, A. M.	2019	Predicting physical activity levels in individuals with schizophrenia through integrated global positioning system and accelerometer data.
Andersen, E.	2018	Physical activity pattern and cardiorespiratory fitness in individuals with schizophrenia compared with a population-based sample.
Andersen, E.	2020	Effect of high-intensity interval training on cardiorespiratory fitness, physical activity and body composition in people with schizophrenia: A randomized controlled trial.
Apiquian, R.	2008	Variations of rest - Activity rhythm and sleep - Wake in schizophrenic patients versus healthy subjects: An actigraphic comparative study.
Baandrup, L.	2015	A validation of wrist actigraphy against polysomnography in patients with schizophrenia or bipolar disorder.
Baandrup, L.	2016	Circadian rest-activity rhythms during benzodiazepine tapering covered by melatonin versus placebo add-on: data derived from a randomized clinical trial.
Barnett, I.	2018	Relapse prediction in schizophrenia through digital phenotyping: a digital study
Bartolomeo, L. A.	2023	The positivity offset theory of anhedonia in schizophrenia: evidence for a deficit in daily life using digital phenotyping.
Beebe, L. H.	2013	Description of physical activity in outpatients with schizophrenia spectrum disorders.
Beebe, L. H.	2013	A pilot study describing physical activity in persons with schizophrenia spectrum disorders (Ssds) after an exercise program.
Bengtsson, J.	2021	Ambulatory Heart Rate Variability in Schizophrenia or Depression: Impact of Anticholinergic Burden and Other Factors.
Ben-Zeev, D.	2016	Mobile Behavioral Sensing for Outpatients and Inpatients With Schizophrenia.

Ben-Zeev, D.	2017	CrossCheck: Integrating Self-Report, Behavioral Sensing, and Smartphone Use to Identify Digital Indicators of Psychotic Relapse.
Ben-Zeev, D.	2017	Use of multimodal technology to identify digital correlates of violence among inpatients with serious mental illness: A pilot study.
Ben-Zeev, D.	2020	Mobile RDoC: Using Smartphones to Understand the Relationship Between Auditory Verbal Hallucinations and Need for Care.
Berle, J. O.	2010	Actigraphic registration of motor activity reveals a more structured behavioural pattern in schizophrenia than in major depression.
Berry, A.	2021	Examining the feasibility, acceptability, validity and reliability of physical activity, sedentary behaviour and sleep measures in people with schizophrenia.
Bioque, M.	2023	Deep brain stimulation and digital monitoring for patients with treatment-resistant schizophrenia and bipolar disorder: A case series.
Blomqvist, M.	2023	Relationship between Physical Activity and Health Outcomes in Persons with Psychotic Disorders after Participation in a 2-Year Individualized Lifestyle Intervention.
Bracht, T.	2012	Comparison of objectively measured motor behavior with ratings of the motor behavior domain of the Bern Psychopathology Scale (BPS) in schizophrenia.
Bracht, T.	2013	Altered cortico-basal ganglia motor pathways reflect reduced volitional motor activity in schizophrenia.
Brobakken, M. F.	2019	A comprehensive cardiovascular disease risk profile in patients with schizophrenia.
Bromundt, V.	2011	Sleep - Wake cycles and cognitive functioning in schizophrenia.
Browne, J.	2016	Work out by walking: A pilot exercise program for individuals with schizophrenia spectrum disorders.
Browne, J.	2021	Targeting physical health in schizophrenia: Results from the Physical Activity Can Enhance Life (PACE-Life) 24
Browne, J.	2023	Virtual group-based walking intervention for persons with schizophrenia: A pilot randomized controlled trial.
Buck, B.	2019	Capturing behavioral indicators of persecutory ideation using mobile technology.
Buck, B.	2019	Relationships between smartphone social behavior and relapse in schizophrenia: A preliminary report.
Buck, B.	2022	The relationship between appraisals of auditory verbal hallucinations and real-time affect and social functioning.
Bueno-Antequera, J.	2018	Sedentary behaviour, physical activity, cardiorespiratory fitness and cardiometabolic risk in psychosis: The PsychiActive project.
Bueno-Antequera, J.	2018	Ideal cardiovascular health and its association with sedentary behaviour and fitness in psychiatric patients. The PsychiActive project.
Cella, M.	2018	Using wearable technology to detect the autonomic signature of illness severity in schizophrenia.
Cella, M.	2019	Blending active and passive digital technology methods to improve symptom monitoring in early psychosis.
Cella, M.	2022	Evaluating the mechanisms of social cognition intervention in schizophrenia: A proof-of-concept trial.
Chen, L. J.	2016	Association between actigraphy-derived physical activity and cognitive performance in patients with schizophrenia.
Chen, L. J.	2022	Associations between daily steps and cognitive function among inpatients with schizophrenia.

Chen, M. D.	2017	A pilot comparative study of one-way versus two-way text message program to promote physical activity among people with severe mental illness.
Chung, K. F.	2018	Correlates of sleep irregularity in schizophrenia.
Chung, K. F.	2020	Subjective-Objective Sleep Discrepancy in Schizophrenia.
Cochran, J. M.	2021	Characterization of activity behavior using a digital medicine system and comparison to medication ingestion in patients with serious mental illness.
Cochran, J. M.	2022	Participant Engagement and Symptom Improvement: Aripiprazole Tablets with Sensor for the Treatment of Schizophrenia.
Cohen, A.	2023	Relapse prediction in schizophrenia with smartphone digital phenotyping during COVID-19
Cohen, A. S.	2022	Natural Language Processing and Psychosis: On the Need for Comprehensive Psychometric Evaluation.
De Girolamo, G.	2020	DAily time use, Physical Activity, quality of care and interpersonal relationships in patients with Schizophrenia spectrum disorders (DiAPASon): An Italian multicentre study.
Deenik, J.	2017	Physical activity and quality of life in long-term hospitalized patients with severe mental illness: A cross-sectional study.
Deenik, J.	2018	Improved psychosocial functioning and quality of life in inpatients with severe mental illness receiving a multidisciplinary lifestyle enhancing treatment. The MULTI study II.
Deenik, J.	2019	Changes in physical and psychiatric health after a multidisciplinary lifestyle enhancing treatment for inpatients with severe mental illness: The MULTI study I.
Dennison, C. A.	2021	Association of genetic liability for psychiatric disorders with accelerometer-assessed physical activity in the UK Biobank.
Depp, C. A.	2019	GPS mobility as a digital biomarker of negative symptoms in schizophrenia: a case control study.
Diamond, R.	2022	The physical activity profiles of patients with persecutory delusions.
Docx, L.	2013	Quantitative psychomotor dysfunction in schizophrenia: A loss of drive, impaired movement execution or both?
Duncan, M. J.	2017	Revisiting the International Physical Activity Questionnaire (IPAQ): Assessing physical activity among individuals with schizophrenia.
Duncan, M. J.	2019	Revisiting the International Physical Activity Questionnaire (IPAQ): Assessing sitting time among individuals with schizophrenia.
Engh, J. A.	2019	Objectively assessed daily steps-not light intensity physical activity, moderate-to-vigorous physical activity and sedentary time-is associated with cardiorespiratory fitness in patients with schizophrenia.
Fang, S. H.	2016	Associations between sleep quality and inflammatory markers in patients with schizophrenia.
Farrow, T. F. D.	2005	Structural brain correlates of unconstrained motor activity in people with schizophrenia.
Farrow, T. F. D.	2006	Modafinil and unconstrained motor activity in schizophrenia: Double-blind crossover placebo-controlled trial.
Farrow, T. F. D.	2009	A neuroanatomical basis for the frequency of discrete spontaneous activities in schizophrenia.
Fasmer, E. E.	2018	Graph theory applied to the analysis of motor activity in patients with schizophrenia and depression.
Faulkner, G.	2006	Validation of a physical activity assessment tool for individuals with schizophrenia.

Firth, J.	2018	The validity and value of self-reported physical activity and accelerometry in people with schizophrenia: A population-scale study of the UK biobank.
Fowler, J. C.	2021	Hummingbird study: Results from an exploratory trial assessing the performance and acceptance of a digital medicine system in adults with schizophrenia, schizoaffective disorder, or first-episode psychosis.
Fulford, D.	2021	Smartphone sensing of social interactions in people with and without schizophrenia.
Gardos, G.	1992	Quantitative assessment of psychomotor activity in patients with neuroleptic-induced akathisia.
Gomes, E.	2014	Effects of a group physical activity program on physical fitness and quality of life in individuals with schizophrenia.
Gomes, E.	2016	Quality of life and physical activity levels in outpatients with schizophrenia.
Gorczynski, P.	2014	Examining strategies to improve accelerometer compliance for individuals living with schizophrenia.
Gorczynski, P.	2014	Examining the efficacy and feasibility of exercise counseling in individuals with schizophrenia: A single-case experimental study.
Gothelf, D.	2002	Weight gain associated with increased food intake and low habitual activity levels in male adolescent schizophrenic inpatients treated with olanzapine.
Grassmann, V.	2017	The relationship between moderate-to-vigorous physical activity and executive function among individuals with schizophrenia: Differences by illness duration.
Heidary, Z.	2021	A Rest Quality Metric Using a Cluster-Based Analysis of Accelerometer Data and Correlation With Digital Medicine Ingestion Data: Algorithm Development.
Henson, P.	2020	Towards clinically actionable digital phenotyping targets in schizophrenia.
Henson, P.	2020	Impact of dynamic greenspace exposure on symptomatology in individuals with schizophrenia.
Henson, P.	2021	Anomaly detection to predict relapse risk in schizophrenia.
Henson, P.	2021	Investigating Associations Between Screen Time and Symptomatology in Individuals With Serious Mental Illness: Longitudinal Observational Study.
He-Yueya, J.	2020	Assessing the relationship between routine and schizophrenia symptoms with passively sensed measures of behavioral stability.
Hofstetter, J. R.	2005	Quality of sleep in patients with schizophrenia is associated with quality of life and coping.
Holmen, T. L.	2019	The Association Between Cardiorespiratory Fitness and Cognition Appears Neither Related to Current Physical Activity Nor Mediated by Brain-Derived Neurotrophic Factor in a Sample of Outpatients With Schizophrenia.
Holt, R. I. G.	2019	Structured lifestyle education for people with schizophrenia, schizoaffective disorder and first-episode psychosis (STEPWISE): randomised controlled trial.
Ifrah, C.	2020	Cognitive insight and autonomic regulation during daily functioning in individuals with schizophrenia.
Janney, C. A.	2013	Sedentary behavior and psychiatric symptoms in overweight and obese adults with schizophrenia and schizoaffective disorders (WAIST Study).
Janney, C. A.	2015	Physical activity and sedentary behavior measured objectively and subjectively in overweight and obese adults with schizophrenia or schizoaffective disorders.
Jerome, G. J.	2009	Physical activity levels of persons with mental illness attending psychiatric rehabilitation programs.

Jongs, N.	2020	A framework for assessing neuropsychiatric phenotypes by using smartphone-based location data.
Juda, M.	2023	Sleep and Rest-Activity Rhythms in Recovering Patients with Severe Concurrent Mental and Substance Use Disorder: A Pilot Study.
Kalisperakis, E.	2023	Smartwatch digital phenotypes predict positive and negative symptom variation in a longitudinal monitoring study of patients with psychotic disorders.
Kammerer, M. K.	2021	Sleep and circadian rhythm disruption predict persecutory symptom severity in day-to-day life: A combined actigraphy and experience sampling study.
Kane, I.	2012	Feasibility of pedometers for adults with schizophrenia: Pilot study.
Kane, J. M.	2013	First experience with a wireless system incorporating physiologic assessments and direct confirmation of digital tablet ingestions in ambulatory patients with schizophrenia or bipolar disorder.
Kas, M. J. H.	2024	Digital behavioural signatures reveal trans-diagnostic clusters of Schizophrenia and Alzheimer's disease patients: Trans-diagnostic clustering of digital biotypes.
Kearns Murphy, C.	2022	Operation recovery: a feasibility study of an 8 week exercise and lifestyle programme within an Irish first episode psychosis service
Kidd, S. A.	2019	Feasibility and outcomes of a multi-function mobile health approach for the schizophrenia spectrum: APP4
Kim, J.	2020	Association of Depression With Functional Mobility in Schizophrenia.
Kimhy, D.	2014	Aerobic fitness and body mass index in individuals with schizophrenia: Implications for neurocognition and daily functioning.
Kishimoto, T.	2008	Antipsychotic-induced hyperprolactinemia inhibits the hypothalamo- pituitary-gonadal axis and reduces bone mineral density in male patients with schizophrenia.
Kluge, A.	2018	Combining actigraphy, ecological momentary assessment and neuroimaging to study apathy in patients with schizophrenia.
Knights, J.	2019	Evaluating digital medicine ingestion data from seriously mentally ill patients with a Bayesian Hybrid Model.
Kodaka, M.	2010	Misalignments of rest-activity rhythms in inpatients with schizophrenia.
Krane-Gartiser, K.	2018	Motor activity patterns in acute schizophrenia and other psychotic disorders can be differentiated from bipolar mania and unipolar depression.
Kruisdijk, F.	2017	Accelerometer-measured sedentary behaviour and physical activity of inpatients with severe mental illness.
Kurebayashi, Y.	2017	Association between altered physical activity and neurocognitive function among people with schizophrenia: A minimum 6
Kurebayashi, Y.	2017	Correlations between physical activity and neurocognitive domain functions in patients with schizophrenia: A cross-sectional study.
Kuula, L.	2022	Adolescent circadian patterns link with psychiatric problems: A multimodal approach.
Lahti, A. C.	2021	Clinical utility of wearable sensors and patient-reported surveys in patients with schizophrenia: Noninterventional, observational study.
Lakhtakia, T.	2022	Smartphone digital phenotyping, surveys, and cognitive assessments for global mental health: Initial data and clinical correlations from an international first episode psychosis study.

Lamichhane, B.	2023	Psychotic Relapse Prediction in Schizophrenia Patients Using a Personalized Mobile Sensing-Based Supervised Deep Learning Model.
Lederman, O.	2017	Modifiable cardiometabolic risk factors in youth with at-risk mental states: A cross-sectional pilot study.
Leutwyler, H.	2014	Associations of Schizophrenia Symptoms and Neurocognition With Physical Activity in Older Adults With Schizophrenia.
Leutwyler, H.	2015	The Impact of a Videogame-Based Pilot Physical Activity Program in Older Adults with Schizophrenia on Subjectively and Objectively Measured Physical Activity.
Liebenthal, E.	2022	Linguistic and non-linguistic markers of disorganization in psychotic illness.
Lindamer, L. A.	2008	Assessment of physical activity in middle-aged and older adults with schizophrenia.
Liu, G.	2019	Assessing the potential of longitudinal smartphone based cognitive assessment in schizophrenia: A naturalistic pilot study.
Mandel, F.	2021	Neural Networks for Clustered and Longitudinal Data Using Mixed Effects Models.
Martanto, W.	2021	Association between wrist wearable digital markers and clinical status in Schizophrenia.
Martin, J.	2001	Actigraphic estimates of circadian rhythms and sleep/wake in older schizophrenia patients.
Martin, J. L.	2005	Older schizophrenia patients have more disrupted sleep and circadian rhythms than age-matched comparison subjects.
Mayeli, A.	2023	Shared and distinct abnormalities in sleep-wake patterns and their relationship with the negative symptoms of Schizophrenia Spectrum Disorder patients.
Meng, Q.	2018	Effects of chlorpromazine on sleep quality, clinical and emotional measures among patients with schizophrenia.
Methapatara, W.	2011	Pedometer walking plus motivational interviewing program for Thai schizophrenic patients with obesity or overweight: A 12
Meyer, N.	2018	Capturing Rest-Activity Profiles in Schizophrenia Using Wearable and Mobile Technologies: Development, Implementation, Feasibility, and Acceptability of a Remote Monitoring Platform.
Minor, K. S.	2022	Personalizing Interventions Using Real-World Interactions: Improving Symptoms and Social Functioning in Schizophrenia With Tailored Metacognitive Therapy.
Moran, E. K.	2024	Loneliness in the Daily Lives of People With Mood and Psychotic Disorders.
Mow, J. L.	2022	Smartphone-based mobility metrics capture daily social motivation and behavior in schizophrenia.
Mulligan, L. D.	2016	High resolution examination of the role of sleep disturbance in predicting functioning and psychotic symptoms in schizophrenia: A novel experience sampling study.
Nadesalingam, N.	2023	The Behavioral Mapping of Psychomotor Slowing in Psychosis Demonstrates Heterogeneity Among Patients Suggesting Distinct Pathobiology.
Narkhede, S. M.	2022	Machine Learning Identifies Digital Phenotyping Measures Most Relevant to Negative Symptoms in Psychotic Disorders: Implications for Clinical Trials.
Nguyen, D. K.	2022	Decision support system for the differentiation of schizophrenia and mood disorders using multiple deep learning models on wearable devices data.
Niendam, T. A.	2018	Enhancing early psychosis treatment using smartphone technology: A longitudinal feasibility and validity study.
Ogasawara, M.	2023	Exploratory Validation of Sleep-Tracking Devices in Patients with Psychiatric Disorders.

Oliva, V.	2023	Patterns of antipsychotic prescription and accelerometer-based physical activity levels in people with schizophrenia spectrum disorders: A multicenter, prospective study.
Orleans-Pobee, M.	2022	Physical Activity Can Enhance Life (PACE-Life): results from a 10-week walking intervention for individuals with schizophrenia spectrum disorders
Osipov, M.	2015	Objective identification and analysis of physiological and behavioral signs of schizophrenia.
Parrish, E. M.	2020	Emotional determinants of life-space through GPS and ecological momentary assessment in schizophrenia: What gets people out of the house?
Peters-Strickland, T.	2016	Usability of a novel digital medicine system in adults with schizophrenia treated with sensor-embedded tablets of aripiprazole.
Pieters, L. E.	2021	Exploring the Relationship between Movement Disorders and Physical Activity in Patients with Schizophrenia: An Actigraphy Study.
Pieters, L. E.	2023	Combining actigraphy and experience sampling to assess physical activity and sleep in patients with psychosis: A feasibility study.
Poon, Y. P. Y.-P.	2018	Delayed sleep-wake phase disorder and delayed sleep-wake phase in schizophrenia: Clinical and functional correlates.
Poyurovsky, M.	2000	Actigraphic monitoring (actigraphy) of circadian locomotor activity in schizophrenic patients with acute neuroleptic-induced akathisia.
Price, G. D.	2022	An unsupervised machine learning approach using passive movement data to understand depression and schizophrenia.
Ranjan, T.	2022	Longitudinal symptom changes and association with home time in people with schizophrenia: An observational digital phenotyping study.
Ransing, R.	2021	Comparison of actigraphy indices among patients with depression and schizophrenia: A preliminary study.
Raugh, I. M.	2020	Geolocation as a Digital Phenotyping Measure of Negative Symptoms and Functional Outcome.
Raugh, I. M.	2021	Digital phenotyping adherence, feasibility, and tolerability in outpatients with schizophrenia.
Reeve, S.	2019	Sleep Disorders in Early Psychosis: Incidence, Severity, and Association with Clinical Symptoms.
Reinertsen, E.	2017	Continuous assessment of schizophrenia using heart rate and accelerometer data.
Reinertsen, E.	2018	Multiscale network dynamics between heart rate and locomotor activity are altered in schizophrenia.
Reshef, A.	2013	The effects of acupuncture treatment on sleep quality and on emotional measures among individuals living with schizophrenia: A pilot study.
Robillard, R.	2015	Ambulatory sleep-wake patterns and variability in young people with emerging mental disorders.
Rodriguez-Ruiz, J. G.	2022	Classification of Depressive and Schizophrenic Episodes Using Night-Time Motor Activity Signal.
Ryu, J.	2020	Outdoor cycling improves clinical symptoms, cognition and objectively measured physical activity in patients with schizophrenia: A randomized controlled trial.
Sano, W.	2012	Enhanced Persistency of Resting and Active Periods of Locomotor Activity in Schizophrenia.
Savage, C. L. G.	2021	Assessing the psychometric properties of the PROMIS sleep measures in persons with psychosis

Scheewe, T. W.	2019	Low physical activity and cardiorespiratory fitness in people with schizophrenia: A comparison with matched healthy controls and associations with mental and physical health.
Servaas, M. N.	2019	Rigidity in motor behavior and brain functioning in patients with schizophrenia and high levels of apathy.
Shamir, E.	2000	Melatonin improves sleep quality of patients with chronic schizophrenia.
Sharpe, J. K.	2006	Accelerometry is a valid measure of physical inactivity but not of energy expended on physical activity in people with schizophrenia.
Shin, S.	2016	Activity monitoring using a mHealth device and correlations with psychopathology in patients with chronic schizophrenia.
Skeldon, A. C.	2022	Extracting Circadian and Sleep Parameters from Longitudinal Data in Schizophrenia for the Design of Pragmatic Light Interventions.
Smit, M. M. C.	2022	Evaluating the implementation of a multidisciplinary lifestyle intervention for people with severe mental illness in sheltered housing: effectiveness-implementation hybrid randomised controlled trial.
Soundy, A.	2007	Psychometric Properties of the 7-Day Physical Activity Recall Questionnaire in Individuals with Severe Mental Illness
Strauss, G. P.	2022	Validation of accelerometry as a digital phenotyping measure of negative symptoms in schizophrenia.
Stubbs, B.	2017	Physical activity ameliorates the association between sedentary behavior and cardiometabolic risk among inpatients with schizophrenia: A comparison versus controls using accelerometry.
Stubbs, B.	2017	Relationship between objectively measured sedentary behavior and cognitive performance in patients with schizophrenia vs controls.
Thomas-Brown, P. G. L.	2018	Risperidone provides better improvement of sleep disturbances than haloperidol therapy in schizophrenia patients with Cannabis-Positive urinalysis.
Thonon, B.	2022	A Group Intervention for Motivational Deficits: Preliminary Investigation of a Blended Care Approach Using Ambulatory Assessment.
Torous, J.	2018	Characterizing the clinical relevance of digital phenotyping data quality with applications to a cohort with schizophrenia.
Tous-Espelosin, M.	2021	Clinical, physical, physiological, and cardiovascular risk patterns of adults with schizophrenia: CORTEX-SP study: Characterization of adults with schizophrenia.
Tseng, V. W. S.	2020	Using behavioral rhythms and multi-task learning to predict fine-grained symptoms of schizophrenia.
Umbricht, D.	2020	Deep Learning-Based Human Activity Recognition for Continuous Activity and Gesture Monitoring for Schizophrenia Patients With Negative Symptoms.
Vancampfort, D.	2017	Lower cardiorespiratory fitness is associated with more time spent sedentary in first episode psychosis: A pilot study.
Vancampfort, D.	2019	Validity and correlates of the International Physical Activity Questionnaire in first-episode psychosis.
von Kanel, S.	2022	Measuring catatonia motor behavior with objective instrumentation.
Wainberg, M.	2021	Association of accelerometer-derived sleep measures with lifetime psychiatric diagnoses: A cross-sectional study of 89
Walther, S.	2009	Increased motor activity in cycloid psychosis compared to schizophrenia.
Walther, S.	2009	Quantitative motor activity differentiates schizophrenia subtypes.
Walther, S.	2009	Objectively measured motor activity in schizophrenia challenges the validity of expert ratings.

Walther, S.	2010	Higher motor activity in schizophrenia patients treated with olanzapine versus risperidone.
Walther, S.	2011	Alterations of white matter integrity related to motor activity in schizophrenia.
Walther, S.	2011	Resting state cerebral blood flow and objective motor activity reveal basal ganglia dysfunction in schizophrenia.
Walther, S.	2014	Less structured movement patterns predict severity of positive syndrome, excitement, and disorganization.
Walther, S.	2015	The longitudinal course of gross motor activity in schizophrenia - within and between episodes.
Walther, S.	2015	Physical activity in schizophrenia is higher in the first episode than in subsequent ones.
Walther, S.	2022	Low physical activity is associated with two hypokinetic motor abnormalities in psychosis.
Wang, J.	2012	Both physical activity and food intake are associated with metabolic risks in patients with schizophrenia.
Waters, F.	2011	Daily variations in sleep-wake patterns and severity of psychopathology: A pilot study in community-dwelling individuals with chronic schizophrenia.
Wichniak, A.	2011	Actigraphic monitoring of activity and rest in schizophrenic patients treated with olanzapine or risperidone.
Williams, J.	2019	'Walk this way': Results from a pilot randomised controlled trial of a health coaching intervention to reduce sedentary behaviour and increase physical activity in people with serious mental illness.
Wirz-Justice, A.	2001	Disturbed circadian rest-activity cycles in schizophrenia patients: an effect of drugs?
Wisniewski, H.	2019	Using a Smartphone App to Identify Clinically Relevant Behavior Trends via Symptom Report, Cognition Scores, and Exercise Levels: A Case Series.
Wulff, K.	2012	Sleep and circadian rhythm disruption in schizophrenia.
Zarbo, C.	2022	Assessing adherence to and usability of Experience Sampling Method (ESM) and actigraph in patients with Schizophrenia Spectrum Disorder: A mixed-method study
Zarbo, C.	2023	Ecological monitoring of physical activity, emotions and daily life activities in schizophrenia: the DiAPAson study.
Zhou, J.	2022	Predicting Psychotic Relapse in Schizophrenia With Mobile Sensor Data: Routine Cluster Analysis.
Zlatintsi, A.	2022	E-Prevention: Advanced Support System for Monitoring and Relapse Prevention in Patients with Psychotic Disorders Analyzing Long-Term Multimodal Data from Wearables and Video Captures.

Supplementary Table 4. *Completed PRISMA checklist*

Section and Topic	Item #	Checklist item	Location where item is reported
**TITLE**			
Title	1	Identify the report as a systematic review.	Pg 1
**ABSTRACT**			
Abstract	2	See the PRISMA 2020 for Abstracts checklist.	
**INTRODUCTION**			
Rationale	3	Describe the rationale for the review in the context of existing knowledge.	Pg 4
Objectives	4	Provide an explicit statement of the objective(s) or question(s) the review addresses.	Pg 4
**METHODS**			
Eligibility criteria	5	Specify the inclusion and exclusion criteria for the review and how studies were grouped for the syntheses.	Pg 5
Information sources	6	Specify all databases, registers, websites, organisations, reference lists and other sources searched or consulted to identify studies. Specify the date when each source was last searched or consulted.	Pg 4
Search strategy	7	Present the full search strategies for all databases, registers and websites, including any filters and limits used.	Pg 4/5
Selection process	8	Specify the methods used to decide whether a study met the inclusion criteria of the review, including how many reviewers screened each record and each report retrieved, whether they worked independently, and if applicable, details of automation tools used in the process.	Pg 5
Data collection process	9	Specify the methods used to collect data from reports, including how many reviewers collected data from each report, whether they worked independently, any processes for obtaining or confirming data from study investigators, and if applicable, details of automation tools used in the process.	Pg 5
Data items	10a	List and define all outcomes for which data were sought. Specify whether all results that were compatible with each outcome domain in each study were sought (e.g. for all measures, time points, analyses), and if not, the methods used to decide which results to collect.	Pg 5/6
	10b	List and define all other variables for which data were sought (e.g. participant and intervention characteristics, funding sources). Describe any assumptions made about any missing or unclear information.	Pg 5/6
Study risk of bias assessment	11	Specify the methods used to assess risk of bias in the included studies, including details of the tool(s) used, how many reviewers assessed each study and whether they worked independently, and if applicable, details of automation tools used in the process.	Pg 6
Effect measures	12	Specify for each outcome the effect measure(s) (e.g. risk ratio, mean difference) used in the synthesis or presentation of results.	n/a
Synthesis methods	13a	Describe the processes used to decide which studies were eligible for each synthesis (e.g. tabulating the study intervention characteristics and comparing against the planned groups for each synthesis (item #5)).	n/a
	13b	Describe any methods required to prepare the data for presentation or synthesis, such as handling of missing summary statistics, or data conversions.	Pg 6
	13c	Describe any methods used to tabulate or visually display results of individual studies and syntheses.	Pg 6
	13d	Describe any methods used to synthesize results and provide a rationale for the choice(s). If meta-analysis was performed, describe the	Pg 6

Section and Topic	Item #	Checklist item	Location where item is reported
		model(s), method(s) to identify the presence and extent of statistical heterogeneity, and software package(s) used.	
	13e	Describe any methods used to explore possible causes of heterogeneity among study results (e.g. subgroup analysis, meta-regression).	n/a
	13f	Describe any sensitivity analyses conducted to assess robustness of the synthesized results.	n/a
Reporting bias assessment	14	Describe any methods used to assess risk of bias due to missing results in a synthesis (arising from reporting biases).	n/a
Certainty assessment	15	Describe any methods used to assess certainty (or confidence) in the body of evidence for an outcome.	n/a
**RESULTS**			
Study selection	16a	Describe the results of the search and selection process, from the number of records identified in the search to the number of studies included in the review, ideally using a flow diagram.	Pg 6/7
	16b	Cite studies that might appear to meet the inclusion criteria, but which were excluded, and explain why they were excluded.	
Study characteristics	17	Cite each included study and present its characteristics.	Pg 7
Risk of bias in studies	18	Present assessments of risk of bias for each included study.	Pg 14/ supplementary file
Results of individual studies	19	For all outcomes, present, for each study: (a) summary statistics for each group (where appropriate) and (b) an effect estimate and its precision (e.g. confidence/credible interval), ideally using structured tables or plots.	n/a
Results of syntheses	20a	For each synthesis, briefly summarise the characteristics and risk of bias among contributing studies.	Pg 8 -14
	20b	Present results of all statistical syntheses conducted. If meta-analysis was done, present for each the summary estimate and its precision (e.g. confidence/credible interval) and measures of statistical heterogeneity. If comparing groups, describe the direction of the effect.	Pg 8-14
	20c	Present results of all investigations of possible causes of heterogeneity among study results.	n/a
	20d	Present results of all sensitivity analyses conducted to assess the robustness of the synthesized results.	n/a
Reporting biases	21	Present assessments of risk of bias due to missing results (arising from reporting biases) for each synthesis assessed.	n/a
Certainty of evidence	22	Present assessments of certainty (or confidence) in the body of evidence for each outcome assessed.	n/a
**DISCUSSION**			
Discussion	23a	Provide a general interpretation of the results in the context of other evidence.	Pg 15-16
	23b	Discuss any limitations of the evidence included in the review.	Pg 16
	23c	Discuss any limitations of the review processes used.	Pg 16
	23d	Discuss implications of the results for practice, policy, and future research.	Pg 16/17

Section and Topic	Item #	Checklist item	Location where item is reported
**OTHER INFORMATION**			
Registration and protocol	24a	Provide registration information for the review, including register name and registration number, or state that the review was not registered.	Pg 4
	24b	Indicate where the review protocol can be accessed, or state that a protocol was not prepared.	Pg 4
	24c	Describe and explain any amendments to information provided at registration or in the protocol.	Supplementary file
Support	25	Describe sources of financial or non-financial support for the review, and the role of the funders or sponsors in the review.	Pg 17
Competing interests	26	Declare any competing interests of review authors.	Pg 17
Availability of data, code and other materials	27	Report which of the following are publicly available and where they can be found: template data collection forms; data extracted from included studies; data used for all analyses; analytic code; any other materials used in the review.	Pg 18

SupplementaryTable 5. *List of all features and behaviours used in the studies*

Behaviour	Feature	Number of times used
Environment	Ambient sound	5
	Ambient light	8
Location or mobility	Distance travelled	19
	Distance from home	11
	Time spent at home/primary location	20
	Time spent away from home/ primary location	3
	Number of unique or significant locations	17
	Location entropy	9
	Flight length	8
	Flight duration	9
	Duration spent stationary	6
	Radius of gyration	5
Phone use	Number of incoming/outgoing calls	15
	Duration of incoming/outgoing calls	12
	Call responsiveness/reciprocity	5
	Number of incoming/outgoing text messages	16
	Length of incoming/outgoing text messages	4
	Text responsiveness/reciprocity	4
	Number of screen active periods	3
	Duration of screen active/unlock periods	11
	Number of screen checks	2
	Phone use during sleep	1
	Number of apps used	3
Physical activity	Mean acceleration/activity count	31
	Total acceleration/ activity count	28
	Movement index	9
	Duration of uninterrupted mobility periods (MIP)	8
	Duration of still or sedentary periods	41
	Duration of active periods	24
	Duration of light activity periods	26
	Duration of moderate activity periods	23
	Duration of vigorous activity periods	21
	Duration of moderate or vigorous activity periods	22
	Step count	41
	Distance walked on foot	1
	Duration of walking time	11
	Energy expenditure	13
	Rest-activity rhythm L5	6
	Rest-activity rhythm M10	6
	Rest-activity rhythm relative amplitude	9
	Rest-activity rhythm intraday stability (IS)	9
	Rest-activity rhythm intraday variability (IV)	10
	Rest-activity rhythm entropy	6
Physiology	Heart rate	14
	Heart rate variability	6
	EDA/skin conductance	3
	Skin temperature	1
Sleep	Sleep start time	17
	Sleep end time	16
	Sleep midpoint	2
	Duration of sleep/ total sleep time (TST)	50
	Duration of time in bed	10
	Sleep wake ratio/ proportion of time asleep	6
	Number of wake events during sleep	19
	Duration of wake after sleep onset (WASO)	23
	Duration of sleep onset latency (SOL)	25
	Sleep efficiency (SE)	32

	Sleep fragmentation	5
	Sleep stages (REM/light etc)	4
	Duration of daytime sleep	3
Sociability	Number of social interactions or conversations	8
	Duration of social interactions or conversations	14
	Quality of social interactions	1
Circadian rhythm	Mobility routine	7
	Physical activity	22
	Phone use	1
	Sleep	20
	Physiology	1
	Sociability	1
Data quantity	Missing GPS data	4
	Missing phone use data	1
	Device wear time	7
	Amount of data collected	2

Supplementary Table 6. *List of all features by device type*

Device type	Feature	n
Accelerometers	sleep_duration_tst	35
	pa_duration_still_sedentary	29
	sleep_efficiency	29
	pa_duration_lpa	24
	pa_mean_acceleration_activity_count	23
	pa_total_acceleration_activity_count	23
	sleep_onset_latency	23
	sleep_waso	21
	pa_duration_mvpa	19
	circadian_sleep	18
	pa_duration_mpa	18
	circadian_pa	16
	pa_duration_vpa	16
	sleep_wake_events_arousals	16
	sleep_start_time	13
	sleep_end_time	12
	pa_duration_active	10
	pa_energy_expenditure	10
	pa_step_count	10
	sleep_tib	10
	pa_duration_uninterrupted_mobility_mip	8
	pa_movement_index	8
	pa_ra_iv	7
	pa_ra_is	6
	pa_ra_ra	6
	sleep_fragmentation	5
	sleep_wake_ratio_prop_asleep	4
	env_ambient_light	3
	pa_duration_walking	3
	pa_ra_l5	3
	pa_ra_m10	3
	sleep_daytime_duration	3
	pa_ra_entropy	2
	sleep_midpoint	2
	sleep_stages_duration_type	1
Other Wearable	phys_heart_rate	8
	pa_step_count	7

	pa_duration_still_sedentary	6
	pa_duration_mpa	5
	pa_duration_vpa	5
	circadian_pa	3
	pa_duration_active	3
	pa_duration_mvpa	3
	pa_mean_acceleration_activity_count	3
	pa_ra_entropy	3
	pa_total_acceleration_activity_count	3
	phys_eda_skin_conductance	3
	phys_heart_rate_variability	3
	sleep_duration_tst	3
	pa_duration_lpa	2
	pa_energy_expenditure	2
	pa_ra_is	2
	pa_ra_iv	2
	pa_ra_l5	2
	pa_ra_m10	2
	pa_ra_ra	2
	sleep_efficiency	1
	sleep_onset_latency	1
	sleep_stages_duration_type	1
	sleep_wake_events_arousals	1
	sleep_waso	1
Pedometer	pa_step_count	11
	pa_duration_walking	1
	pa_energy_expenditure	1
Smartphone	loc_time_spent_primary_loc_home	20
	loc_distance_travelled	19
	loc_no_unique_locations	17
	phone_no_incoming_outgoing_text	16
	phone_no_incoming_outgoing_calls	15
	phone_duration_incoming_outgoing_calls	12
	loc_distance_from_home_primary	11
	phone_duration_screen_active_unlock	11
	loc_flight_duration	9
	loc_location_entropy	9
	loc_flight_length	8
	sleep_duration_tst	8
	circadian_mobility_routine	7
	loc_duration_stationary	6
	pa_duration_active	6
	env_ambient_sound	5
	loc_radius_of_gryation	5
	pa_duration_still_sedentary	5
	phone_call_responsiveness_reciprocity	5
	env_ambient_light	4
	loc_no_flights	4
	pa_mean_acceleration_activity_count	4
	phone_app_usage	4
	phone_length_incoming_outgoing_texts	4
	phone_text_responsiveness	4
	loc_time_away_from_primary	3
	sleep_end_time	3
	sleep_start_time	3

	circadian_pa	2
	pa_duration_walking	2
	phone_no_screen_active_periods	2
	phone_screen_checks	2
	circadian_sleep	1
	env_environment_type	1
	pa_movement_index	1
	pa_ra_entropy	1
	pa_ra_is	1
	pa_ra_iv	1
	pa_ra_l5	1
	pa_ra_m10	1
	pa_ra_ra	1
	pa_total_acceleration_activity_count	1
	sleep_wake_events_arousals	1
Smartwatch or Commercial Fitness Band	pa_step_count	13
	phys_heart_rate	6
	pa_duration_active	5
	pa_duration_walking	5
	sleep_duration_tst	4
	phys_heart_rate_variability	3
	sleep_efficiency	2
	sleep_stages_duration_type	2
	sleep_wake_ratio_prop_asleep	2
	circadian_pa	1
	circadian_sleep	1
	env_ambient_light	1
	pa_duration_still_sedentary	1
	pa_mean_acceleration_activity_count	1
	pa_total_acceleration_activity_count	1
	phone_no_screen_active_periods	1
	sleep_end_time	1
	sleep_onset_latency	1
	sleep_start_time	1
	sleep_wake_events_arousals	1
	sleep_waso	1

Supplementary Table 7. *Summary of prediction model and prognostic factor studies.*

Aim	Ariticle	Device	Outcome	Behaviours	Model	Key findings
**Predicting ASM/EMA scores**	Adler 2022	Smartphone	EMA score for sleep and stress	Location/mobility, phone use, physical activity, sleep, sociability, missing data	Gradient boosted regression trees	FNN autoencoder achieved median sensitivity of 0.25 and specificity of 0.88 at predicting anomalies. Features that had greatest effect varied between participants who relapsed.
	He-Yueya et al 2020	Smartphone	Overall EMA score	Environment, phone use, physical activity, sleep, sociability, circadian rhythm	Gradient boosted regression trees	Models predicting EMA scores from stability index 7 days in advance MAE 2.266, 14 days in advance MAE 2.553. Previous EMA score was best performing model MAE 2.061
	Mandel et al 2023	Smartphone	EMA scores for depression and anxiety	Location/mobility, physical activity, circadian rhythm	Generalised neural network mixed model	GNMM with two layers had lowest MSPE in predicting anxiety (0.062) and depression scores (0.428)
	Mulligan et al 2016	Actigraph	EMA scores for psychotic symptoms and functioning	Sleep	Mixed effects regression	Sleep duration and quality measures predicted next day functioning and next day psychotic symptoms, after adjusting for baseline PANSS score.
	Tseng et al 2020	Smartphone	EMA scores - 10 domains	Environment, location/mobility, phone use, physical activity, sleep, sociability, circadian rhythm	Multiple multi-task and single-task learning algorithms, and m-SVR to predict EMA scores. K-means algorithm	m-SVR with RBF kernel model had lowest median RMSE (0.314) in test data for predicting scores. Phone usage had highest feature weight for predicting all EMA domains except thinking clearly and feeling calm.

					to cluster feature weights	
**Identifying/predicting anomalies**	Adler et al 2020	Smartphone	Behavioural anomalies prior to relapse	Location/mobility, phone use, physical activity, sleep, circadian rhythm, sociability	Encoder-decoder neural networks	Median sensitivity 0.25 (IQR 0.15-1.00) & specificity 0.88 (IQR 0.14-0.96) for FNN AD algorithm at detecting anomalies
	Barnett et al 2018	Smartphone	Behavioural anomalies prior to relapse	Location/mobility, phone use, circadian rhythm, sociability	Multivariate timeseries anomaly detection method	Overall mobility features had highest median number of anomalies. For individuals who relapsed the rate of anomalies within 2 weeks before relapse was 71% higher than in other weeks
	Buck et al 2019	Smartphone	Behavioural anomalies prior to relapse	Phone use, sociability	Generalised estimated equations	Daily outgoing call duration, no. of incoming and outgoing text messages lower on average in days preceding relapse, but did vary when day split up into 4 periods.
	Cohen et al 2023	Smartphone	Behavioural anomalies prior to relapse	Location/mobility, phone use, sociability, sleep	Multivariate timeseries anomaly detection method	Model combining passive and active symptom data performed better than active symptom data alone
	Henson et al 2021	Smartphone	Behavioural anomalies prior to relapse	Location/mobility, phone use, sociability, sleep	Multivariate timeseries anomaly detection method	Mobility, sociability, sleep and screen time features had higher rates of anomalies than EMA or cognitive test scores.
	Zlatinsi et al et al 2022	Smartwatch	Behavioural anomalies prior to relapse	Physical activity, physiology, sleep	Transformers, FNN, CNN, GRU	CNN model highest median ROC-AUC (0.61) and PR-AUC (0.76) for personalised models. Model including accelerometer and HR data

						had highest PR-AUC (0.76) but HR only model had highest ROC-AUC (0.52)
**Identifying/predicting relapse**	Lamichhane et al 2023	Smartphone	Relapse (7 days)	Environment, location/mobility, phone use, physical activity, sociability	LSTM based deep learning model	Model achieved F2 score of 0.52 in test data, higher than encoder-decoder model
	Zhou et al 2022	Smartphone	Relapse (7 days)	Environment, location/mobility, phone use, physical activity, sleep, sociability	Balanced RF for prediction, GMM amd PAM for deriving cluster features	Model with baseline passive features and clustering features highest F2 score (0.23). Most significant features included distance travelled, conversation and sound level metrics, along with clustering features.
**Classification of psychosis or schizophrenia**	Narkhede et al 2022	Smartphone and wrist wearable	Classification of psychosis and healthy controls	Location/mobility, physical activity	RF, k-nearest neighbours, logistic regression	Random forest models outperformed others, AUC ranged from 0.818 to 0.845. Passive features identified as significant were distance from home and acceleration metrics.
	Nguyen et al 2022	Actigraph	Classification of schizophrenia, mood disorder and healthy controls	Physical activity	Convolutional and recurrent neural networks	On average CNN models performed with greater accuracy (0.74 ± 0.02 vs 0.68 ± 0.05) and precision (0.70 ± 0.02 vs 0.66 ± 0.04) than RNN models
	Osipov et al 2015	Wearable adhesive patch	Classification of schizophrenia from healthy controls	Physical activity, physiology	Support vector machine with mRMR for feature selection	Model with combination of HR (mean and MSE), physical activity (mode and SD) and transfer entropy features had highest accuracy (95.3%), sensitivity

						(98%), specificity (91.1%) and AUC (0.99)
	Price et al 2022	Actigraph	classification of schizophrenia, major depressive disorder and healthy controls	Physical activity, circadian rhythm	Unsupervised clustering algorithm (UMAP)	Subjects with common diagnosis tended to cluster together. SHAP values showed SZ subjects had decreased dirurnal regularity and activity in early morning hours.
	Reinertsen et al 2017	Wearable adhesive patch	Classification of schizophrenia from healthy controls	Physical activity, physiology	Support vector machine	Model with HR and activity data for 8-day window highest AUC (0.96) vs HR (0.90) or activity (0.89) data alone. 11 most predictive features in model identified.
	Reinertsen et al 2018	Wearable adhesive patch	Classification of schizophrenia from healthy controls	Physical activity, physiology	Support vector machine with mSNR for feature selection	4 models achieved AUC of 1.00 in test data, combinations of MSNR, MSE and MTE
	Rodrigues-Ruiz et al 2022	Actigraph	classification of schizophrenia, major depressive disorder and healthy controls	Physical activity	RF, k-nearest neighbours, logistic regression	Model with activity data from nighttime (00:00-05:59 am) had highest overall accuracy (98.24%) compared to morning (87.97%), afternoon (80.92%) or evening (89.84%) periods.

Supplementary Table 8. *Risk of bias assessment for prognostic factor studies, using QUIPS*

Author	Year	Title	Study Participation	Study Attrition	PF measurement	Outcome measurement	Study confounding	Statistical analysis and reporting	Overall
Adler, D. A., et al.	2020	Predicting Early Warning Signs of Psychotic Relapse From Passive Sensing Data: An Approach Using Encoder-Decoder Neural Networks.	Moderate	Low	Low	Low	High	Low	High
Adler, D. A., et al.	2022	Machine learning for passive mental health symptom prediction: Generalization across different longitudinal mobile sensing studies.	High	High	Moderate	Moderate	High	Low	High
Barnett, I., et al.	2018	Relapse prediction in schizophrenia through digital phenotyping: a digital study	High	Low	Low	Low	High	Low	High
Buck, B., et al.	2019	Relationships between smartphone social behavior and relapse in schizophrenia: A preliminary report.	Moderate	Low	Moderate	Low	High	Low	High
Cohen, A., et al.	2023	Relapse prediction in schizophrenia with smartphone digital phenotyping during COVID-19	Moderate	High	High	Low	Moderate	Low	High
Henson, P., et al.	2021	Anomaly detection to predict relapse risk in schizophrenia.	High	Moderate	Moderate	Low	High	Low	High
He-Yueya, J., et al.	2020	Assessing the relationship between routine and schizophrenia symptoms with passively sensed measures of behavioral stability.	Moderate	Low	Moderate	Low	High	Low	High
Mulligan, L. D., et al.	2016	High resolution examination of the role of sleep disturbance in predicting functioning and psychotic symptoms in schizophrenia: A novel experience sampling study.	Moderate	Low	Moderate	Low	High	Low	High

Narkhede, S. M., et al.	2022	Machine Learning Identifies Digital Phenotyping Measures Most Relevant to Negative Symptoms in Psychotic Disorders: Implications for Clinical Trials.	Moderate	High	Moderate	Low	High	Low	High
Osipov, M., et al.	2015	Objective identification and analysis of physiological and behavioral signs of schizophrenia.	High	High	Moderate	High	High	Low	High
Price, G. D., et al.	2022	An unsupervised machine learning approach using passive movement data to understand depression and schizophrenia.	High	High	High	High	High	Low	High
Reinertsen, E., et al.	2017	Continuous assessment of schizophrenia using heart rate and accelerometer data.	High	High	High	High	High	Low	High
Reinertsen, E., et al.	2018	Multiscale network dynamics between heart rate and locomotor activity are altered in schizophrenia.	High	High	Moderate	High	High	Low	High
Rodriguez-Ruiz, J. G., et al.	2022	Classification of Depressive and Schizophrenic Episodes Using Night-Time Motor Activity Signal.	High	High	High	High	High	Low	High
Tseng, V. W. S., et al.	2020	Using behavioral rhythms and multi-task learning to predict fine-grained symptoms of schizophrenia.	High	Low	Moderate	Low	High	Low	High
Zlatintsi, A., et al.	2022	E-Prevention: Advanced Support System for Monitoring and Relapse Prevention in Patients with Psychotic Disorders Analyzing Long-Term Multimodal Data from Wearables and Video Captures.	Moderate	High	Low	Low	High	Low	High

Supplementary Table 9. *Risk of bias assessment for prediction model studies, using PROBAST*

Author	Year	Title	Participants	Predictors	Outcome	Analysis	Overall
Zhou, J., et al.	2022	Predicting Psychotic Relapse in Schizophrenia With Mobile Sensor Data: Routine Cluster Analysis.	Low	Low	Low	High	High
Nguyen, D. K., et al.	2022	Decision support system for the differentiation of schizophrenia and mood disorders using multiple deep learning models on wearable devices data.	Unclear	Unclear	Unclear	Unclear	Unclear
Lamichhane, B., et al.	2023	Psychotic Relapse Prediction in Schizophrenia Patients Using a Personalized Mobile Sensing-Based Supervised Deep Learning Model.	Unclear	Low	Low	Unclear	Unclear
